# Mediterranean Diet, Physical Activity, and Bone Health in Older Adults: A Secondary Analysis of a Randomized Clinical Trial

**DOI:** 10.1001/jamanetworkopen.2025.3710

**Published:** 2025-04-08

**Authors:** Héctor Vázquez-Lorente, Jesús F. García-Gavilán, Sangeetha Shyam, Jadwiga Konieczna, J. Alfredo Martínez, Vicente Martín-Sánchez, Montserrat Fitó, Miguel Ruiz-Canela, Indira Paz-Graniel, Ariadna Curto, Diego Martinez-Urbistondo, José Antonio de Paz Fernández, Dora Romaguera, Nancy Babio, Jordi Salas-Salvadó

**Affiliations:** 1 ANUT-DSM (Alimentaciò, Nutrició Desenvolupament i Salut Mental), Departament de Bioquímica i Biotecnologia, Universitat Rovira i Virgili, Reus, Spain; 2 Centro de Investigación Biomédica en Red Fisiopatología de la Obesidad y la Nutrición, Institute of Health Carlos III, Madrid, Spain; 3 Institut d’Investigació Sanitària Pere Virgili, Reus, Spain; 4 Grupo NUTRECOR (Nutritional Epidemiology and Cardiovascular Physiopathology), Health Research Institute of the Balearic Islands, Palma de Mallorca, Spain; 5 Department of Nutrition, Food Sciences, and Physiology, Center for Nutrition Research, University of Navarra, Pamplona, Spain; 6 Precision Nutrition and Cardiometabolic Health Program, IMDEA Food (Institutos Madrileño de Estudios Avanzados), Campus of International Excellence, Universidad de Autonoma Madrid—Spanish National Research Council, Madrid, Spain; 7 Departamento de Medicina y Endocrinología, Universidad de Valladolid, Valladolid, Spain; 8 Institute of Biomedicine, University of León, León, Spain; 9 Centro de Investigación Biomédica en Red, Epidemiología y Salud Pública, Instituto de Salud Carlos III, Madrid, Spain; 10 Cardiovascular Risk and Nutrition Unit, Hospital del Mar Research Institute, Barcelona, Spain; 11 Department of Preventive Medicine and Public Health, Instituto de Investigación Sanitària de Navarra, University of Navarra, Pamplona, Spain

## Abstract

**Importance:**

Understanding the effects of diet and physical activity on bone health may help to mitigate concomitant weight loss– and age-related bone deterioration.

**Objective:**

To evaluate the effects of a weight-loss lifestyle intervention on bone mineral density (BMD), bone mineral content (BMC), and low BMD prevalence.

**Design, Setting, and Participants:**

The 3-year, parallel-group Prevención con Dieta Mediterránea–Plus (PREDIMED-Plus) randomized clinical trial was conducted at recruiting centers in Spain from October 2013 to December 2016. Men and women (aged 55-75 years) with metabolic syndrome and overweight or obesity, as well as access to dual-energy x-ray absorptiometry (DXA) (from 4 centers in Navarra, Mallorca, Reus, and León), were included in this prespecified secondary analysis. The analysis was conducted from September 1 to October 30, 2024.

**Intervention:**

Participants were randomly allocated (1:1) to the intervention or the control. The intervention group followed an energy-reduced Mediterranean diet and increased physical activity, whereas the control group was advised to follow an ad libitum Mediterranean diet with no physical activity promotion.

**Main Outcomes and Measures:**

BMD (total femur, lumbar spine [L1-L4], and femoral trochanter) and total BMC were measured using DXA at baseline and at 1 and 3 years of follow-up. T scores from BMD variables were determined, and osteopenia or osteoporosis status was defined as low BMD. Linear and logistic 2-level mixed models were used for main analyses including participants lost to follow-up. Intention-to-treat analysis, completers case analysis, and sensitivity analysis were additionally performed.

**Results:**

This study included 924 older adults (mean [SD] age, 65.1 [5.0] years; 454 women [49.1%] and 470 men [50.9%]). Significant differences in mean changes between groups were observed, with an overall 3-year intervention increase in the intervention group (n = 460) compared with the control group (n = 464) in lumbar spine (L1-L4) BMD (between-group differences, −0.1 [95% CI, –0.8 to 0.8] g/cm^2^ after 1 year and 0.9 [95% CI, 0.1-1.8] g/cm^2^ after 3 years; overall *P* = .05). This protective association was observed for women (between-group differences, −0.1 [95% CI, –1.3 to 1.1] g/cm^2^ after 1 year and 1.8 [95% CI, 0.6-2.9] g/cm^2^ after 3 years; overall *P* = .005) but not for men. No overall 3-year intervention effect on total BMC and low BMD prevalence was observed.

**Conclusions and Relevance:**

In the PREDIMED-Plus trial, an energy-reduced Mediterranean diet and physical activity lifestyle intervention mitigated weight loss– and age-related BMD decline among older women with metabolic syndrome compared with conventional ad libitum Mediterranean diet recommendations. Weight-loss lifestyle interventions with longer follow-up are warranted in the future to confirm these results in relation to bone health.

**Trial Registration:**

ISRCTN Registry Identifier: ISRCTN89898870

## Key Points

**Question:**

What are the effects of concomitant weight loss resulting from a healthy weight-loss lifestyle intervention on age-related bone deterioration?

**Findings:**

This secondary analysis of a randomized clinical trial included 924 older adults with metabolic syndrome and overweight or obesity. An energy-reduced Mediterranean diet and physical activity promotion, compared with advice to follow an ad libitum Mediterranean diet, produced significant beneficial effects on bone mineral density, especially at the lumbar level, over 3 years among women.

**Meaning:**

These findings suggest that a weight-loss lifestyle intervention based on an energy-reduced Mediterranean diet and physical activity promotion delivered to older women at risk of bone disturbances may be a feasible strategy for potentially mitigating the effects of concomitant weight loss on age-related decline in bone mineral density.

## Teaser

This secondary analysis of a randomized clinical trial examines effects of an energy-reduced Mediterranean diet and physical activity promotion, compared with ad libitum Mediterranean diet alone, on bone health over 3 years among older adults with metabolic syndrome and overweight or obesity.

## Introduction

Low bone mineral density (BMD) and total bone mineral content (BMC) result in osteoporotic fractures, increasing morbidity, diminishing quality of life, and increasing mortality rates.^1^ Given the concurrent global increase in the prevalence of osteoporosis and population aging,^2^ the health care sector, along with public and policy institutions, will face substantial challenges that demand the implementation of effective measures to mitigate the burden.^3^

Preventive recommendations for reducing osteoporotic fractures emphasize the importance of maintaining a balanced diet and engaging in regular physical activity.^2^ These measures are especially crucial for individuals with obesity and older individuals, who have greater susceptibility to osteoporotic fractures.^4^ Because weight loss is a frequent measure to manage obesity-related conditions,^5^ understanding the modifiable effects of diet and physical activity on bone health is instrumental in developing strategies to reduce the effect of weight loss on age-related bone deterioration.^6^

Adherence to a nutrient-dense, healthy dietary pattern during energy reduction may help preserve BMD and total BMC.^7^ The Mediterranean diet offers potential benefits for bone health across all age groups.^6^ Although energy-reduced diets aimed at inducing weight loss in obese older adults remain a subject of debate, primarily due to the concomitant reduction in BMD and total BMC,^8^ incorporating physical activity for that purpose could alleviate and mitigate some of these adverse effects,^9^ although it does not result in bone health improvement.^10^

In the era of more effective antiobesity treatments, weight-loss lifestyle interventions aimed at promoting BMD and total BMC maintenance and mitigating osteoporotic risk and fracture during periods of weight loss are thus urgently needed.^11^ The Prevención con Dieta Mediterránea–Plus (PREDIMED-Plus) randomized clinical trial presents an opportunity to assess bone health outcomes of a multifactorial intervention that included consumption of an energy-reduced Mediterranean diet, physical activity promotion, and behavioral support among older adults with metabolic syndrome and overweight or obesity.^12,13^ This study aimed to evaluate the effect of the PREDIMED-Plus weight-loss lifestyle intervention on changes in age-related BMD, total BMC, and low BMD prevalence over 3 years of follow-up.

## Methods

### Study Design

The PREDIMED-Plus randomized clinical trial was conducted across 23 Spanish centers. The primary objective was to assess the effect of an energy-reduced Mediterranean diet, increased physical activity, and behavioral support on the prevention of cardiovascular disease and total body weight loss.^14^ This prespecified secondary analysis evaluated the effect of the PREDIMED-Plus intervention on intermediate outcomes, specifically bone-related variables (ie, BMD, total BMC, and low BMD prevalence). Detailed information regarding the study protocol can be found online^15^ and in previous publications^12,13^; the trial protocol and statistical analysis plan are available in Supplement 1. Ethical approval was granted by the institutional review boards of all participating centers, and written informed consent was obtained from all participants. The study adhered to the Consolidated Standards of Reporting Trials (CONSORT) reporting guideline.

### Participants

The study population consisted of community-dwelling adults (aged 55-75 years) with overweight (body mass index [BMI], 27.0-29.9; calculated as weight in kilograms divided by height in meters squared) or obesity (BMI, 30.0-39.9)^16^ who met at least 3 criteria for metabolic syndrome. To identify metabolic syndrome, the following updated criteria from the International Diabetes Federation, the American Heart Association, and the National Heart, Lung, and Blood Institute were used: hypertension, plasma triglycerides (≥150 mg/dL [to convert to mmol/L, multiply by 0.0113]), plasma high-density lipoprotein cholesterol (<40 mg/dL for men or <50 mg/dL for women [to convert to mmol/L, multiply by 0.0259]), fasting blood glucose (>100 mg/dL [to convert to mmol/L, multiply by 0.0555]), and central obesity (≥102 cm for men or ≥88 cm for women).^17^ Individuals with preexisting cardiovascular disease at baseline were excluded. From October 2013 to December 2016, a total of 6874 eligible participants were randomized in a 1:1 ratio to either the control group or the intervention group, with randomization stratified by center, sex, and age. Couples living in the same household were randomized together. For this secondary analysis, data were drawn from a subset of participants from the 4 centers that had access to dual-energy x-ray absorptiometry (DXA) scanners (Navarra, Mallorca, Reus, and León) and conducted BMD and total BMC measurements. Of these, older adults with metabolic syndrome who had complete baseline data were analyzed.

### Intervention

Participants in the control group were instructed to follow an ad libitum traditional Mediterranean diet, based on previous considerations for the PREDIMED study,^18^ without energy restriction or specific physical activity recommendations. In contrast, participants in the intervention group were prescribed a Mediterranean diet with a 30% energy reduction, and they received counseling to gradually increase their physical activity to meet the World Health Organization (WHO) recommendation of 150 minutes of moderate to vigorous physical activity per week for adults aged 65 or older. Specifically, participants in the intervention group were encouraged to walk for a minimum of 45 minutes per day, 6 days per week; and to engage in strength, flexibility, and balance exercises 3 days per week, along with 30- to 40-minute sessions of resistance training 2 days per week.^19^ Behavioral and motivational strategies, including self-monitoring, goal-setting, and problem-solving, were also implemented in the intervention group to promote the sustainable adoption of dietary and lifestyle modifications.^19^

### Bone Assessment

BMD variables and total BMC were assessed using DXA bone densitometers (DXA Lunar Prodigy Primo and Lunar iDXA; GE Healthcare) at baseline and at 1 and 3 years of follow-up. The study targeted clinically significant bone sites for BMD analysis, including the total femur, lumbar spine (L1-L4), and femoral trochanter. BMD of the femoral region was measured on the nondominant side. T scores for these 3 sites were subsequently calculated based on reference values for the Spanish adult population, adjusted for sex, age, weight, and height. Due to the insufficient number of patients with osteoporosis, statistical analyses considering the typical 3 categories of BMD (normal, osteopenia, and osteoporosis) could not be performed without introducing bias. Therefore, a dichotomous variable, referred to as *low BMD status* (including osteopenia and osteoporosis in the same variable), was used. This variable was defined using BMD T scores and a modification of the WHO T-score cutoffs for each site as low BMD status (1) when T scores were −1 or less and as normal BMD status (0) when T scores were greater than −1.^20^

### Covariate Assessments

Sociodemographic and lifestyle information regarding age, sex, educational level, marital status, smoking status, and medical history was collected through administered questionnaires. Anthropometric variables, such as weight and height, were assessed using calibrated scales and wall-mounted stadiometers, respectively. BMI was also calculated.^2^ Population questionnaires validated in Spanish were used to collect data on total leisure-time physical activity,^21^ sedentary behavior,^22^ adherence to the energy-reduced Mediterranean diet,^23^ and total energy intake.^24^

### Statistical Analysis

These analyses were prespecified and performed according to the previously published statistical analysis plan (Supplement 1). The sample size for PREDIMED-Plus was calculated only for the primary outcomes of the trial (for the combined cardiovascular end point, the required sample size was 3000 in each group; for body weight loss, the required sample size was 337 in each group),^13,14^ not for the intermediate outcomes reported herein. A post hoc power analysis was conducted to assess whether a sample size of 450 participants per study group was sufficient to detect the observed small effect size (Cohen *d* = 0.2) using a significance level of α = .05 for total femur BMD, obtaining a power of 92%.

Baseline characteristics of the study cohort are presented, by intervention group and sex, as means with SDs for continuous variables and numbers with percentages for categorical variables. The unpaired *t* test and the χ^2^ test were used for continuous and categorical variables, respectively.

A priori interaction analyses were conducted for BMD variables and total BMC by baseline categories of age (<65 or ≥65 years), sex (male or female), smoking habits (current, former, or never), diabetes (yes or no), physical activity (less than the median or median or greater), and BMI (overweight or obesity) using the likelihood ratio test. An interaction term between the intervention group, time, and each potential effect modifier was included within multivariable-adjusted models. All analyses were further stratified by sex as an interaction was detected, and main analyses were conducted for participants with available baseline data and follow-up data at 1 and 3 years. For participants lost to follow-up (intention-to-treat analyses), imputation was performed to include all missing data for BMD variables and total BMC, with the missing values handled using the multiple imputation by chained equations method (Stata “mi” command) using fully conditional specification (imputed datasets = 20; seed = 1234). Moreover, participants with missing variables over 1 and 3 years of follow-up were additionally excluded to perform a completers case analysis.

Two-level linear mixed models to assess intervention group effects on changes in BMD and total BMC and 2-level logistic mixed models to evaluate intervention group effects on low BMD prevalence were conducted, with random intercepts at the cluster family (because couples from the same household were randomized together) and individual participant levels. In both cases, interaction terms between the intervention group and time, age (years), sex (male or female), and recruiting center (Navarra, Mallorca, Reus, or León) were included as fixed effects in the basic models. Baseline educational level (primary or less, secondary, or college), marital status (single, divorced or separated, married, or widowed), smoking status (current, former, or never), diabetes prevalence (yes or no), hypertension prevalence (yes or no), hypercholesterolemia prevalence (yes or no), BMI, physical activity (metabolic equivalents in minutes per day), sedentary time (hours per day), calcium or vitamin D medication or supplementation (or both) (yes or no), use of osteoporotic drugs (yes or no), adherence to the energy-restricted Mediterranean diet (0-17 points), and daily energy intake (kilocalories per day) were additionally included as fixed effects in the multivariable-adjusted models. Sex (male or female) was excluded as a covariate in the analyses that were stratified by sex. Linear mixed models are presented as intragroup mean changes and intergroup mean differences, whereas the results of logistic mixed models are presented as odds ratios (ORs), along with their corresponding 95% CIs in both cases. As sensitivity analyses, participants consuming calcium or vitamin D medication or supplementation (or both) at all time points (n = 188) were excluded.

All statistical analyses were conducted with Stata/SE, version 14.2 (StataCorp LLC), using the PREDIMED-Plus study dataset updated to December 19, 2023. Graphs were plotted using GraphPad Prism, version 9.0 (GraphPad Software). Statistical significance was defined as *P* ≤ .05 (2-tailed). Data analysis was conducted from September 1 to October 30, 2024.

## Results

Of the 1384 older adults in the PREDIMED-Plus study with DXA scan data available, 924 with metabolic syndrome were included in this secondary analysis (Figure). Baseline characteristics of these 924 participants (mean [SD] 65.1 [5.0] years) were balanced by study group (464 in the control group and 460 in the intervention group) and by sex (454 women [49.1%] and 470 men [50.9%]) (Table 1). eTable 1 in Supplement 2 presents the number of study participants with and without data on bone variables from centers having access to DXA devices. eTable 2 in Supplement 2 shows the baseline characteristics of participants selected for DXA measurements compared with those not selected from the total cohort.

**Figure.**
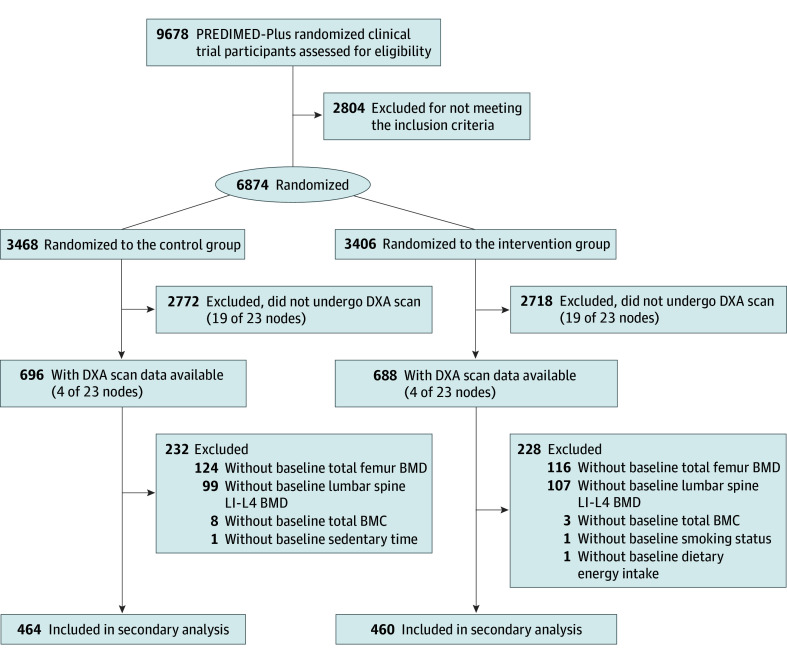
Flowchart of the Study Population BMC indicates bone mineral content; BMD, bone mineral density; DXA, dual-energy x-ray absorptiometry; PREDIMED-Plus, Prevención con Dieta Mediterránea–Plus.

**Table 1.** Baseline Characteristics of Participants by Group and Sex

Characteristic	Control group (n = 464)		Intervention group (n = 460)		*P* value^a^
	Men (n = 233)	Women (n = 231)	Men (n = 237)	Women (n = 223)	
**Sociodemographic**					
Age, mean (SD), y	63.8 (5.3)	66.5 (4.0)	63.2 (5.4)	66.3 (4.1)	.17
Educational level, No. (%)					
Primary or less	99 (42.5)	141 (61.0)	95 (40.1)	145 (65.3)	.93
Secondary	79 (33.9)	60 (26.0)	82 (34.6)	51 (23.0)	
College	55 (23.6)	30 (13.0)	55 (25.3)	26 (11.7)	
Marital status, No. (%)					
Single, divorced, or separated	24 (10.3)	32 (14.0)	36 (15.2)	38 (17.1)	.04
Married	205 (88.0)	162 (70.1)	191 (80.6)	140 (63.1)	
Widowed	4 (1.7)	37 (16.0)	10 (4.2)	44 (19.8)	
Disease presence or medication use at recruitment, No. (%)					
Type 2 diabetes	60 (25.8)	55 (23.8)	60 (25.3)	57 (25.7)	.82
Hypertension	196 (84.1)	186 (80.5)	192 (81.0)	172 (77.5)	.22
Hypercholesterolemia	153 (65.7)	178 (77.0)	144 (60.8)	153 (68.9)	.03
Medication use					
Insulin or other antidiabetic drugs	50 (21.5)	42 (18.2)	45 (19.0)	42 (18.9)	.72
Antihypertensive agents	189 (81.1)	184 (79.7)	179 (75.5)	165 (74.3)	.04
Statins or other hypolipidemic drugs	112 (48.1)	127 (55.0)	106 (44.7)	114 (51.4)	.26
Calcium or vitamin D medication or supplementation (or both)	4 (1.7)	26 (11.3)	4 (1.7)	25 (11.3)	.92
Osteoporotic drugs	4 (1.7)	19 (8.2)	3 (1.3)	16 (7.2)	.55
**Lifestyle**					
Physical exercise, mean (SD), METs/min/d	469.2 (401.5)	322.5 (287.6)	403.5 (351.6)	301.7 (275.1)	.06
Sedentary time, mean (SD), h/d	6.1 (1.9)	5.9 (5.7)	6.0 (1.8)	5.8 (1.7)	.30
Smoking status, No. (%)					
Current smoker	33 (14.2)	21 (65.4)	50 (21.1)	13 (5.9)	.49
Former smoker	154 (66.1)	59 (25.6)	141 (59.5)	54 (24.3)	
Never smoker	46 (19.7)	151 (65.4)	46 (19.4)	155 (69.8)	
Anthropometry					
BMI, mean (SD)	31.9 (3.1)	33.1 (3.6)	32.7 (3.0)	33.0 (3.5)	.10
**Dietary**					
Adherence to energy-restricted Mediterranean diet, mean (SD), points (0-17)	8.0 (2.5)	8.9 (2.7)	8.0 (2.5)	8.6 (2.6)	.53
Energy intake, mean (SD), kcal/d	2607 (592)	2323 (602)	2569 (556)	2276 (587)	.31
**Bone**					
BMD, mean (SD), g/cm^2^					
Total femur	1.1 (0.1)	1.0 (0.1)	1.1 (0.1)	1.0 (0.1)	.81
Lumbar spine (L1-L4)	1.0 (0.1)	1.1 (0.2)	1.0 (0.1)	1.1 (0.2)	.77
Femoral trochanter	0.9 (0.1)	0.8 (0.1)	0.9 (0.1)	0.8 (0.1)	.84
Total BMC, mean (SD), g	3030.1 (389.6)	2173.4 (297.3)	3052.4 (380.9)	2179.9 (295.0)	.46

Abbreviations: BMC, bone mineral content; BMD, bone mineral density; BMI, body mass index (calculated as weight in kilograms divided by height in meters squared); MET, metabolic equivalent.

^a^ *P* values for intergroup differences by study group in the overall population were calculated with the Pearson χ^2^ test or unpaired *t* test, as appropriate.

Interaction analyses for the DXA variables and multiple relevant baseline variables are illustrated in the eFigure in Supplement 2. Significant interactions were found by sex for all BMD variables (eFigure A-C in Supplement 2) and by smoking and categories of BMI for lumbar spine (L1-L4) BMD (eFigure B and C in Supplement 2).

Total body weight (in kilograms) decreased 3.3% (3.5% in men and 3.0% in women) and 3.0% (3.4% in men and 2.5% in women) over 1 and 3 years, respectively, in the intervention group compared with 0.4% (0.3% in men and 0.6% in women) and 0.7% (0.5% in men and 0.9% in women) in the control group. Overall 3-year differences in changes in total body weight between groups were significant (between-group difference, –2.8 kg [95% CI, –3.3 to –2.2 kg] after 1 year and –2.2 kg [95% CI, –2.7 to –1.6 kg] after 3 years; overall *P* < .001). Table 2 and eTable 3 in Supplement 2 present the effects of the PREDIMED-Plus intervention on BMD variables and total BMC over 3 years of follow-up in the overall population and by sex. After adjusting for multiple covariates, significant differences in mean changes between groups were observed. An overall 3-year intervention increase was observed for the intervention group compared with the control group for lumbar spine (L1-L4) BMD in the overall population (between-group difference, −0.1 [95% CI, –0.8 to 0.8] g/cm^2^ after 1 year and 0.9 [95% CI, 0.1-1.8] g/cm^2^ after 3 years; overall *P* = .05) (Table 2 and eTable 3 in Supplement 2), and this protective association was also observed in women (between-group difference, −0.1 [95% CI, –1.3 to 1.1] g/cm^2^ after 1 year and 1.8 [95% CI, 0.6-2.9] g/cm^2^ after 3 years; overall *P* = .005) (Table 2 and eTable 3 in Supplement 2) but not in men. Moreover, no overall intervention group effect on low BMD prevalence was shown over 3 years of follow-up (Table 3 and eTable 4 in Supplement 2).

**Table 2.** Effect of Intervention on BMD Variables and Total BMC Content Over 3 Years by Overall Population and Sex

Variable	Mean change (95% CI)^a^			*P* value^b^
	Control group	Intervention group	Difference	
**Overall (N = 924; 464 vs 460)**				
BMD, g/cm^2^^c^				
Total femur				
Year 1 vs baseline	–0.1 (–0.5 to 0.3)	–0.3 (–0.7 to 0.1)	–0.2 (–0.8 to 0.4)	.44
Year 3 vs baseline	–1.0 (–1.4 to –0.6)	–1.4 (–1.8 to –1.0)	–0.4 (–1.0 to 0.2)	
Lumbar spine (L1-L4)				
Year 1 vs baseline	0.5 (–0.1 to 1.1)	0.5 (–0.1 to 1.1)	–0.1 (–0.8 to 0.8)	.05
Year 3 vs baseline	–0.1 (–0.6 to 0.6)	0.9 (0.3-1.5)	0.9 (0.1-1.8)	
Femoral trochanter				
Year 1 vs baseline	–0.2 (–0.7 to 0.4)	0.1 (–0.5 to 0.6)	0.2 (–0.5 to 1.0)	.83
Year 3 vs baseline	–0.8 (–1.3 to –0.3)	–0.7 (–1.2 to –0.1)	0.2 (–0.6 to 0.9)	
Total BMC, g				
Year 1 vs baseline	–10.3 (–18.8 to –1.9)	–13.0 (–21.7 to –4.2)	–2.6 (–14.8 to 9.5)	.91
Year 3 vs baseline	–18.5 (–26.9 to –10.0)	–19.7 (–28.6 to –10.8)	–1.2 (–13.5 to 11.1)	
**Men (n = 470; 233 vs 237)**				
BMD, g/cm^2^^c^				
Total femur BMD				
Year 1 vs baseline	–0.2 (–0.8 to 0.4)	0.1 (–0.5 to 0.7)	0.3 (–0.5 to 1.1)	.07
Year 3 vs baseline	–0.6 (–1.1 to 0.1)	–1.2 (–1.8 to –0.6)	–0.7 (–1.5 to 0.1)	
Lumbar spine (L1-L4)				
Year 1 vs baseline	0.8 (0.1-1.6)	0.9 (0.1-1.7)	0.1 (–1.1 to 1.2)	.98
Year 3 vs baseline	1.3 (0.6-2.1)	1.4 (0.6-2.2)	0.1 (–1.0 to 1.2)	
Femoral trochanter				
Year 1 vs baseline	–0.4 (–1.1 to 0.4)	0.8 (–0.1 to 1.7)	1.1 (0.1-2.2)	.06
Year 3 vs baseline	–0.4 (–1.2 to 0.3)	–0.4 (–1.2 to 0.4)	0.1 (–1.1 to 1.1)	
Total BMC, g				
Year 1 vs baseline	–11.0 (–23.5 to 1.5)	–4.4 (–17.0 to 8.2)	6.6 (–11.2 to 24.4)	.64
Year 3 vs baseline	–12.6 (–25.1 to –0.1)	–14.5 (–27.4 to –1.6)	–1.9 (–19.9 to 16.0)	
**Women (n = 454; 231 vs 223)**				
BMD, g/cm^2^^c^				
Total femur				
Year 1 vs baseline	–0.1 (–0.5 to 0.5)	–0.8 (–1.4 to –0.3)	–0.8 (–1.6 to –0.1)	.08
Year 3 vs baseline	–1.5 (–2.1 to –1.0)	–1.6 (–2.2 to –1.0)	–0.1 (–0.9 to 0.7)	
Lumbar spine (L1- L4)				
Year 1 vs baseline	0.1 (–0.7 to 0.9)	–0.4 (–1.8 to 1.1)	–0.1 (–1.3 to 1.1)	.005
Year 3 vs baseline	–1.3 (–2.2 to –0.5)	0.4 (–0.5 to –0.5)	1.8 (0.6-2.9)	
Femoral trochanter				
Year 1 vs baseline	0.1 (–0.6 to 0.8)	–0.7 (–1.5 to 0.1)	–0.8 (–1.9 to 0.2)	.12
Year 3 vs baseline	–1.2 (–1.9 to –0.5)	–0.9 (–1.7 to –0.2)	0.3 (–0.8 to 1.3)	
Total BMC, g				
Year 1 vs baseline	–9.6 (–20.8 to 1.5)	–22.9 (–34.8 to –13.5)	–13.2 (–29.5 to 3.1)	.23
Year 3 vs baseline	–24.7 (–35.9 to –13.5)	–25.7 (–37.8 to –13.5)	–0.9 (–17.5 to 15.6)	

Abbreviations: BMC, bone mineral content; BMD, bone mineral density; PREDIMED-Plus, Prevención con Dieta Mediterránea–Plus.

^a^ Two-level linear mixed models were fitted with random intercepts at the cluster family (because couples from the same household were randomized together) and individual participant levels to assess intervention group effects on changes in BMD variables and total BMC measured repeatedly over time (at each follow-up visit and for the overall follow-up period). Interaction terms between the intervention group and time, age (years), sex (male or female), recruiting center (Navarra, Mallorca, Reus, or León), baseline educational level (primary or less, secondary, or college), marital status (single, divorced or separated, married, or widowed), smoking status (current, former, or never), diabetes prevalence (yes or no), hypertension prevalence (yes or no), hypercholesterolemia prevalence (yes or no), body mass index, physical activity (metabolic equivalents in minutes per day), sedentary time (hours per day), calcium or vitamin D medication or supplementation (or both) (yes or no), use of osteoporotic drugs (yes or no), adherence to the energy-restricted Mediterranean diet (0-17 points), and daily energy intake (kilocalories per day) were included as fixed effects in the multivariable-adjusted models. Sex (male or female) was excluded as a covariate in the analyses stratified for sex.

^b^ Represents the intervention group effects assessed for the overall follow-up period. Significance was set at *P ≤* .05.

^c^ Expressed as multiples of 10^−2^ (×10^−2^).

**Table 3.** Effect of Intervention on Low BMD Prevalence Over 3 Years by Overall Population and Sex^a^

Variable	Control group, OR (95% CI)	Intervention group, OR (95% CI)	Overall *P* value^b^
**Overall (N = 924; 464 vs 460)**			
Total femur BMD			
Year 1 vs baseline	1 [Reference]	2.2 (0.6-7.4)	.13
Year 3 vs baseline	1 [Reference]	3.9 (1.1-14.5)	
Lumbar spine (L1-L4) BMD			
Year 1 vs baseline	1 [Reference]	0.6 (0.2-2.5)	.59
Year 3 vs baseline	1 [Reference]	1.8 (0.4-7.4)	
Femoral trochanter BMD			
Year 1 vs baseline	1 [Reference]	0.5 (0.2-1.7)	.13
Year 3 vs baseline	1 [Reference]	2.3 (0.7-7.1)	
**Men (n = 470; 233 vs 237)**			
Total femur BMD			
Year 1 vs baseline	1 [Reference]	2.2 (0.6-7.4)	.12
Year 3 vs baseline	1 [Reference]	3.9 (1.1-14.5)	
Lumbar spine (L1-L4) BMD			
Year 1 vs baseline	1 [Reference]	0.6 (0.2-2.5)	.37
Year 3 vs baseline	1 [Reference]	1.8 (0.4-7.4)	
Femoral trochanter BMD			
Year 1 vs baseline	1 [Reference]	0.5 (0.2-1.7)	.08
Year 3 vs baseline	1 [Reference]	2.3 (0.7-7.1)	
**Women (n = 454;** 231 vs 223**)**			
Total femur BMD			
Year 1 vs baseline	1 [Reference]	2.1 (0.7-6.6)	.43
Year 3 vs baseline	1 [Reference]	1.3 (0.4-4.0)	
Lumbar spine (L1-L4) BMD			
Year 1 vs baseline	1 [Reference]	0.6 (0.1-2.5)	.69
Year 3 vs baseline	1 [Reference]	0.6 (0.2-2.2)	
Femoral trochanter BMD			
Year 1 vs baseline	1 [Reference]	2.2 (0.8-6.3)	.23
Year 3 vs baseline	1 [Reference]	2.1 (0.7-5.9)	

Abbreviations: BMD, bone mineral density; OR, odds ratio.

^a^ Two-level logistic mixed models were fitted with random intercepts at the cluster family (because couples from the same household were randomized together) and individual participant levels to assess the intervention group effect on low BMD prevalence. BMD was considered low for all participants with a T score less than −1 (osteopenia or osteoporosis) in every BMD measure. Interaction terms between the intervention group and time, age (years), sex (male or female), recruiting center (Navarra, Mallorca, Reus, or León), baseline educational level (primary or less, secondary, or college), marital status (single, divorced or separated, married, or widowed), smoking status (current, former, or never), diabetes prevalence (yes or no), hypertension prevalence (yes or no), hypercholesterolemia prevalence (yes or no), body mass index, physical activity (metabolic equivalents in minutes per day), sedentary time (hours per day), calcium or vitamin D medication or supplementation (or both) (yes or no), use of osteoporotic drugs (yes or no), adherence to the energy-restricted Mediterranean diet (0-17 points), and daily energy intake (kilocalories per day) were included in the multivariable-adjusted model. Sex (male or female) was excluded as a covariate in the analyses stratified for sex.

^b^ Represents the intervention group effects assessed for the overall follow-up period. Significance was set at *P ≤* .05.

An overall 3-year intervention increase in total femur BMD (between-group difference, –0.6 [95% CI, –1.9 to 0.7] g/cm^2^ after 1 year and 1.0 [95% CI, –0.3 to 2.3] g/cm^2^ after 3 years; overall *P* = .05), lumbar spine (L1-L4) BMD (between-group difference, 0.2 [95% CI,–1.7 to 2.2] g/cm^2^ after 1 year and 2.2 [95% CI, 0.3-4.2] g/cm^2^ after 3 years; overall *P* = .05), and femoral trochanter BMD (between-group difference, –0.5 [95% CI, –1.8 to 0.9] g/cm^2^ after 1 year and 1.4 [95% CI, 0.1-2.8] g/cm^2^ after 3 years; overall *P* = .01) was observed among women in the intervention group compared with the control group after intention-to-treat analysis (eTable 5 in Supplement 2). Results of the completers case analysis also indicated an increase in lumbar spine (L1-L4) BMD among women in the intervention group during the 3-year period (between-group difference, 0.2 [95% CI, –1.7 to 2.2] g/cm^2^ after 1 year and 1.6 [95% CI, 0.1-3.1] g/cm^2^ after 3 years; overall *P* = .03) (eTable 7 in Supplement 2). In both cases, no overall intervention group effect on low BMD prevalence was shown over 3 years of follow-up (eTables 6 and 8 in Supplement 2).

eTables 9 and 10 in Supplement 2 present the results of sensitivity analyses to check the robustness of the findings related to DXA variables after excluding individuals taking calcium or vitamin D medication or supplementation (or both) at all time points. The results remained consistent with the main analyses presented in Table 2, although the overall 3-year intervention effect was also significant for total BMC in women (between-group difference, –25.5 [95% CI, –44.6 to –6.3] g after 1 year and –1.8 [95% CI, –21.1 to –17.6] g after 3 years; overall *P* = .02) and was attenuated on lumbar spine (L1-L4) BMD in the overall population (eTable 9 in Supplement 2).

## Discussion

In this study, an intensive weight-loss lifestyle intervention combining an energy-reduced Mediterranean diet with physical activity, compared with following an ad libitum Mediterranean diet, produced beneficial effects on BMD at the lumbar level over 3 years among older women with metabolic syndrome and overweight or obesity. The main results were also in agreement with the completers case analysis and with the analysis that excluded participants taking calcium or vitamin D medication or supplementation (or both). In the intention-to-treat analysis, the intervention showed greater additional beneficial effects at the femoral level in women. However, no effect on total BMC and low BMD prevalence was shown over 3 years of intervention. These findings support the use of weight-loss lifestyle interventions based on an energy-reduced Mediterranean diet and physical activity promotion for older women at risk of bone disturbances as a feasible strategy to preserve the effects of possible concomitant weight loss on age-related decreases in BMD decline.

Current evidence highlights the potential for weight loss to induce bone loss and increase fracture risk. Clinical trials involving older adults with overweight or obesity have shown that intentional dietary weight loss without concomitant exercise results in BMD reductions.^25^ Furthermore, weight loss through hypocaloric diets, even when combined with exercise, reduces BMD decline among older adults with obesity over 1 year.^26^ When examining the optimal exercise modality for preserving BMD during weight loss induced through a hypocaloric diet among older adults with obesity, neither resistance training nor aerobic training has been shown to prevent BMD losses.^27^ Along this line, 2021 meta-analyses of randomized clinical trials have indicated that resistance exercise is the most effective to attenuate bone losses during weight loss induced by calorie-restricted diets,^28^ although exercise training appears to preserve total BMC rather than BMD.^29^

In a 1-year weight-loss lifestyle intervention involving older adults with obesity and fragility, exercise training attenuated weight loss–induced bone losses, yet no additional effects were observed among those combining a hypocaloric diet with exercise training.^30^ In a study by Villareal et al,^31^ participants were randomized to a hypocaloric diet that provided adequate protein, calcium, and vitamin D, suggesting that even well-structured weight-loss hypocaloric diets may not be sufficient to maintain bone health in older populations; however, the dietary intervention used in that study was not well detailed. Regarding the Look AHEAD trial, the largest long-term study focused on hypocaloric dietary weight loss and increased physical activity on BMD and total BMC to date, greater bone loss was reported for the intervention group compared with the control group in individuals with type 2 diabetes.^32^ In particular, a modest weight loss induced by a low-fat diet and physical activity was associated with (1) modest hip bone losses after 1 year (9% weight loss),^33^ (2) a modest increase in hip bone loss, particularly in men, after 4 years (5% weight loss),^34^ and (3) additional BMD losses, primarily in men (4% weight loss), after 8 years, highlighting the need for BMD preservation and fracture prevention strategies during intentional weight loss. Of note, the study population had diabetes at enrollment, and the hypocaloric diet employed in the Look AHEAD trial was not the Mediterranean diet.^35^

To our knowledge, our study is the first to report a mitigated BMD decline among older women following an energy-reduced healthy diet, increasing physical activity, and receiving behavioral modification. Specifically, significant differences in mean changes between groups were observed, with beneficial changes in lumbar spine (L1-L4) BMD over 3 years among women in the intervention group compared with the control group, which was also observed in the completers case analysis and in the analysis excluding participants taking calcium or vitamin D medication or supplementation. In the intention-to-treat analysis, an additional beneficial effect of the intervention on total femur BMD and femoral trochanter BMD was also shown over 3 years in women. Interestingly, women seemed to benefit most from the intervention. The observed sex differences may be secondary to the fact that women have increased BMD values at the lumber spine compared with men and also are more prone to bone changes accompanying excess weight than men.^36^ Moreover, menopause itself is a risk factor for osteoporosis, and estrogen loss in women at menopause may lower lumbar spine BMD.^37^ The results observed for lumbar spine (L1-L4) BMD in this study should, however, be interpreted with caution, because possible age-related accumulation of calcium deposits in the vertebral region could influence lumbar spine (L1-L4) BMD DXA measurements.^38^ It is also important to note that the effect size observed for these differences may, in fact, be attenuated, because the control group also adhered to a healthy Mediterranean diet but under ad libitum conditions. Lower rates of osteoporosis have been reported in Mediterranean regions compared with their European counterparts.^39^ Adopting a Mediterranean-style eating pattern and context may have modest beneficial effects on mitigating bone deterioration,^40^ contrasting with the proinflammatory dietary patterns typically associated with worsened bone health.^41^

### Strengths and Limitations

Strengths of our study include the multicenter clinical trial design, large sample size, and relatively long follow-up period. Another strength is the repeated measurements of BMD and total BMC using DXA.

However, we acknowledge that this study has limitations. First, our findings are based on a subsample of individuals participating in a randomized clinical trial who agreed to participate in DXA measurements, although the main characteristics of these participants are similar to those randomized in the whole PREDIMED-Plus cohort. Second, the generalizability of our results may be limited to populations other than those studied, where changes in bone health parameters may be more or less evident. Third, there were some losses to follow-up, resulting in missing DXA data. Fourth, femoral neck BMD data were not obtained in DXA measurements; however, to address this limitation, we additionally included total femur BMD in our analysis, because it encompasses information from the femoral neck, thereby mitigating this limitation to some extent. Fifth, the PREDIMED-Plus trial was not designed to evaluate the effect of the intervention on bone-related variables as primary outcomes.

## Conclusions

In this secondary analysis of a randomized clinical trial, weight loss achieved through a modest hypocaloric Mediterranean diet combined with physical activity produced beneficial effects on BMD, especially at the lumbar level, beyond those observed with ad libitum Mediterranean diet recommendations alone, over 3 years among older women with metabolic syndrome. As such, the beneficial positive effects in women observed from this weight-loss lifestyle intervention hold considerable promise and these results are of potential public health and clinical importance, given the high prevalence of osteoporotic fractures in aged populations with excess weight, especially women, and their substantial burden on public health systems. Weight-loss lifestyle interventions with longer follow-up are warranted in the future to confirm our results in relation to bone health.

## Notes

Supplement 1.
Trial Protocol and Statistical Analysis Plan

Supplement 2. **eTable 1.** Number of Study Participants With and Without Data on Bone Variables From Centers Having Access to a DXA Device **eTable 2.** Baseline Characteristics of Study Participants Selected and Nonselected for DXA Measurements **eTable 3.** Effect of the PREDIMED-Plus Intervention on BMD Variables and Total BMC Content Over 3 Years in the Overall Population and by Sex **eTable 4.** Effect of Intervention Group on Low BMD Prevalence Over 3 Years in the Overall Population and by Sex **eTable 5.** Effect of the PREDIMED-Plus Intervention on BMD Variables and Total BMC Content Over 3 Years After Multiple Imputation in the Overall Population and by Sex (Intention-to-Treat Analysis) **eTable 6.** Effect of Intervention Group on Low BMD Prevalence Over 3 Years After Multiple Imputation in the Overall Population and by Sex (Intention-to-Treat Analysis) **eTable 7.** Effect of the PREDIMED-Plus Intervention on BMD Variables and Total BMC Content Over 3 Years After Excluding Participants Lost to Follow-Up in the Overall Population and by Sex (Completers Case Analysis) **eTable 8.** Effect of Intervention Group on Low BMD Prevalence Over 3 Years After Excluding Participants Lost to Follow-Up in the Overall Population and by Sex (Completers Case Analysis) **eTable 9.** Effect of the PREDIMED-Plus Intervention on BMD Variables and Total BMC Content Over 3 Years After Excluding Participants Taking Calcium or Vitamin D Medication or Supplementation (or Both) in the Overall Population and by Sex **eTable 10.** Effect of Intervention Group on Low BMD Prevalence Over 3 Years After Excluding Participants Taking Calcium or Vitamin D Medication or Supplementation (or Both) in the Overall Population and by Sex **eFigure.** Interaction for the BMD Variables and Total BMC With Relevant Baseline Variables of the Study

Supplement 3.
Data Sharing Statement
