# Supplementary material for: Mediterranean Diet, Physical Activity, and Bone Health in Older Adults: A Secondary Analysis of a Randomized Clinical Trial
Source: JAMA Netw Open. 2025 Apr 8;8(4):e253710. doi: 10.1001/jamanetworkopen.2025.3710 (PMC11979728; doi:10.1001/jamanetworkopen.2025.3710)
Supplement: Supplement 2. — eTable 1. Number of Study Participants With and Without Data on Bone Variables From Centers Having Access to a DXA Device eTable 2. Baseline Characteristics of Study Participants Selected and Nonselected for DXA Measurements eTable 3. Effect of the PREDIMED-Plus Intervention on BMD Variables and Total BMC Content Over 3 Years in the Overall Population and by Sex eTable 4. Effect of Intervention Group on Low BMD Prevalence Over 3 Years in the Overall Population and by Sex eTable 5. Effect of the PREDIMED-Plus Intervention on BMD Variables and Total BMC Content Over 3 Years After Multiple Imputation in the Overall Population and by Sex (Intention-to-Treat Analysis) eTable 6. Effect of Intervention Group on Low BMD Prevalence Over 3 Years After Multiple Imputation in the Overall Population and by Sex (Intention-to-Treat Analysis) eTable 7. Effect of the PREDIMED-Plus Intervention on BMD Variables and Total BMC Content Over 3 Years After Excluding Participants Lost to Follow-Up in the Overall Population and by Sex (Completers Case Analysis) eTable 8. Effect of Intervention Group on Low BMD Prevalence Over 3 Years After Excluding Participants Lost to Follow-Up in the Overall Population and by Sex (Completers Case Analysis) eTable 9. Effect of the PREDIMED-Plus Intervention on BMD Variables and Total BMC Content Over 3 Years After Excluding Participants Taking Calcium or Vitamin D Medication or Supplementation (or Both) in the Overall Population and by Sex eTable 10. Effect of Intervention Group on Low BMD Prevalence Over 3 Years After Excluding Participants Taking Calcium or Vitamin D Medication or Supplementation (or Both) in the Overall Population and by Sex eFigure. Interaction for the BMD Variables and Total BMC With Relevant Baseline Variables of the Study [file jamanetwopen-e253710-s002.pdf]

Supplemental Online Content

Vázquez-Lorente H, García-Gavilan JF, Shyam S, et al. Mediterranean diet, physical activity, and bone health in older adults: a secondary analysis of a randomized clinical trial. *JAMA Netw Open*. 2025;8(4):e253710. doi:10.1001/jamanetworkopen.2025.3710

eTable 1. Number of Study Participants With and Without Data on Bone Variables from Centers having access to a DXA Device

eTable 2. Baseline Characteristics of Study Participants Selected and Nonselected for DXA Measurements

eTable 3. Effect of the PREDIMED-Plus Intervention on BMD Variables and Total BMC Content Over 3 Years in the Overall Population and by Sex

eTable 4. Effect of Intervention Group on Low BMD Prevalence Over 3 Years in the Overall Population and by Sex

eTable 5. Effect of the PREDIMED-Plus Intervention on BMD Variables and Total BMC Content Over 3 Years After Multiple Imputation in the Overall Population and by Sex (Intention-to-Treat Analysis)

eTable 6. Effect of Intervention Group on Low BMD Prevalence Over 3 Years After Multiple Imputation in the Overall Population and by Sex (Intention-to-Treat Analysis)

eTable 7. Effect of the PREDIMED-Plus Intervention on BMD Variables and Total BMC Content Over 3 Years After Excluding Participants Lost to Follow-Up in the Overall Population and by Sex (Completers Case Analysis)

eTable 8. Effect of Intervention Group on Low BMD Prevalence Over 3 Years After Excluding Participants Lost to Follow-Up in the Overall Population and by Sex (Completers Case Analysis)

eTable 9. Effect of the PREDIMED-Plus Intervention on BMD Variables and Total BMC Content Over 3 Years After Excluding Participants Taking Calcium or Vitamin D Medication or Supplementation (or Both) in the Overall Population and by Sex

eTable 10. Effect of Intervention Group on Low BMD Prevalence Over 3 Years After Excluding Participants Taking Calcium or Vitamin D Medication or Supplementation (or Both) in the Overall Population and by Sex

eFigure. Interaction for the BMD Variables and Total BMC With Relevant Baseline Variables of the Study

This supplemental material has been provided by the authors to give readers additional information about their work.

© 2025 Vázquez-Lorente H et al. *JAMA Network Open*.

eTable 1. Number of Study Participants With and Without Data on Bone Variables from Centers Having Access to a DXA Device[a]

Characteristic	Total	Included[b]	Non-included[c]
	n	n (%)	n (%)
**Recruiting center**			
All centers with available DXA	1384	924 (66.8)	460 (33.2)
Center 3	331	183 (55.2)	148 (44.8)
Center 4	335	293 (87.5)	42 (12.5)
Center 7	460	299 (65.0)	161 (35.0)
Center 23	258	149 (57.8)	109 (42.2)

Abbreviations: BMD, bone mineral density. BMC, bone mineral content. DXA, dual-energy x-ray absorptiometry.

[a]Data are shown as numbers (percentages). The sample of 1384 participants (n=924) after exclusions of participants with missing data on baseline variables) was coming from four out of the 23 recruitment centers, as those four centers were the only centers that had DXA scanners available and conducted BMD and total BMC measurements. In each of these four centers, either all participants or a sub-sample were invited to DXA scans.

[b]Participants with data on body composition were measured with DXA and included in the analysis.

[c]Participants without DXA data were excluded from the analysis.

© 2025 Vázquez-Lorente H et al. *JAMA Network Open*.

eTable 2. Baseline Characteristics of Study Participants Selected and Nonselected for DXA Measurements[a]

Baseline characteristic	Participants, No. (%)		
	Total population (n=6759)[d]	Selected[b] (n=1378)[d]	Non-selected[c] (n=5381)[d]
**Sociodemographic variables**			
Age, mean (SD), y	65.0 (4.9)	65.1 (5.0)	64.9 (4.9)
Women	3285 (48.6)	644 (46.7)	2641 (49.1)
Education level			
Primary or less	3300 (48.8)	723 (52.5)	2577 (47.9)
Secondary	1958 (29.0)	408 (29.6)	1550 (28.8)
College	1501 (22.2)	247 (17.9)	1254 (23.3)
Civil status			
Single, divorced, or separated	888 (13.1)	198 (14.4)	690 (12.8)
Married	5172 (76.5)	1037 (75.2)	4135 (76.9)
Widower	699 (10.3)	143 (10.4)	556 (10.3)
**Disease presence or medication usage at recruitment**			
Type 2 diabetes	2088 (30.9)	364 (26.4)	1724 (32.0)
Hypertension	5673 (83.9)	1151 (83.5)	4522 (84.0)
Hypercholesterolemia	4738 (70.1)	941 (68.3)	3797 (70.6)
Medication use			
Insulin or other antidiabetic drugs	1788 (26.5)	286 (20.8)	1502 (27.9)
Antihypertensive agents	5299 (78.4)	1100 (79.8)	4199 (78.0)
Statins or other hypolipidemic drugs	3503 (51.8)	693 (50.3)	2810 (52.2)
Calcium and/or vitamin D medication/supplementation	576 (8.5)	79 (5.7)	497 (9.2)
Osteoporotic drugs	339 (5.0)	53 (3.9)	286 (5.3)
**Lifestyle variables**			
Physical exercise, mean (SD), METs/min/d	353.2 (329.3)	379.8 (337.5)	346.4 (326.8)
Sedentary time, mean (SD), h/d	6.0 (2.0)	6.0 (1.8)	6.0 (2.0)
Smoking status			
Current smoker	849 (12.6)	178 (12.9)	671 (12.5)
Former smoker	2944 (43.6)	614 (44.6)	2330 (43.3)
Never smoker	2966 (43.9)	586 (42.5)	2380 (44.2)
**Anthropometry**			
BMI, mean (SD), kg/m^2	32.6 (3.5)	32.6 (3.4)	32.6 (3.5)
**Dietary variables**			
Adherence to the erMedDiet, mean (SD), points	8.5 (2.7)	8.4 (2.6)	8.5 (2.7)
Energy intake, mean (SD), kcal/d	2413 (631)	2489 (595)	2393 (639)

Abbreviations: BMD, bone mineral density. BMI, body mass index. DXA, dual-energy x-ray absorptiometry. erMedDiet, an energy-restricted Mediterranean diet. METs, metabolic equivalents.

[a]Data are presented as n (%) or mean (SD) for categorical and continuous variables, respectively.

[b]Participants selected for potential DXA measurements

[c]Participants non-selected for DXA measurements

[d]Only those participants with available data in all variables at baseline were included. Initially, 6874 participants formed the overall population, with 1384 being selected for DXA measurements and 5491 non-selected.

© 2025 Vázquez-Lorente H et al. *JAMA Network Open*.

eTable 3. Effect of the PREDIMED-Plus Intervention on BMD Variables and Total BMC Content Over 3 Years in the Overall Population and by Sex[a]

Overall[b]								
**Variable**	**Basic model**				**Multivariable-adjusted model**			
**Bone variable**	**Changes in control**	**Changes in intervention**	**Mean difference in changes**	**P-value[f]**	**Changes in control**	**Changes in intervention**	**Mean difference in changes**	**P-value[f]**
**Total femur BMD (g/cm²)[e]**								
Year 1 vs baseline	–0.1 [–0.5-0.3]	–0.3 [–0.7-0.1]	–0.2 [–0.8-0.4]	.44	–0.1 [–0.5-0.3]	–0.3 [–0.7-0.1]	–0.2 [–0.8-0.4]	.44
Year 3 vs baseline	–1.0 [–1.4 to –0.6]	–1.4 [–1.8 to –1.0]	–0.4 [–1.0-0.2]		–1.0 [–1.4 to –0.6]	–1.4 [–1.8 to –1.0]	–0.4 [–1.0-0.2]	
**Lumbar spines L1–L4 BMD (g/cm²)[e]**								
Year 1 vs baseline	0.5 [–0.1-1.1]	0.5 [–0.1-1.1]	–0.1 [–0.8-0.8]	.04	0.5 [–0.1-1.1]	0.5 [–0.1-1.1]	–0.1 [–0.8-0.8]	.05
Year 3 vs baseline	–0.1 [–0.6-0.6]	0.9 [0.3-1.5]	0.9 [–0.1-1.8]		–0.1 [–0.6-0.6]	0.9 [0.3-1.5]	0.9 [0.1-1.8]	
**Femoral trochanter BMD (g/cm²)[e]**								
Year 1 vs baseline	–0.2 [–0.7-0.4]	0.1 [–0.5-0.6]	0.2 [–0.5-1.0]	.84	–0.2 [–0.7-0.4]	0.1 [–0.5-0.6]	0.2 [–0.5-1.0]	.83
Year 3 vs baseline	–0.8 [–1.3 to –0.3]	–0.6 [–1.2 to –0.1]	0.2 [–0.6-0.9]		–0.8 [–1.3 to –0.3]	–0.7 [–1.2 to –0.1]	0.2 [–0.6-0.9]	
**Total bone mineral content (g)**								
Year 1 vs baseline	–10.4 [–18.8 to –2.0]	–13.0 [–21.8 to –4.3]	–2.6 [–14.8-9.5]	.91	–10.3 [–18.8 to –1.9]	–13.0 [–21.7 to –4.2]	–2.6 [–14.8-9.5]	.91
Year 3 vs baseline	–18.5 [–26.9 to –10.0]	–19.7 [–28.6 to –10.8]	–1.3 [–13.5-11.0]		–18.5 [–26.9 to –10.0]	–19.7 [–28.6 to –10.8]	–1.2 [–13.5-11.1]	
**Men[c]**								
**Variable**	**Basic model**				**Multivariable-adjusted model**			
**Bone variable**	**Changes in control**	**Changes in intervention**	**Mean difference in changes**	**P-value[f]**	**Changes in control**	**Changes in intervention**	**Mean difference in changes**	**P-value[f]**
**Total femur BMD (g/cm²)[e]**								
Year 1 vs baseline	–0.2 [–0.8-0.4]	0.1 [–0.5-0.7]	0.3 [–0.5-1.1]	.07	–0.2 [–0.8-0.4]	0.1 [–0.5-0.7]	0.3 [–0.5-1.1]	.07
Year 3 vs baseline	–0.6 [–1.1-0.1]	–1.2 [–1.8 to –0.6]	–0.7 [–1.5-0.1]		–0.6 [–1.1-0.1]	–1.2 [–1.8 to –0.6]	–0.7 [–1.5-0.1]	

© 2025 Vázquez-Lorente H et al. *JAMA Network Open*.

**Lumbar spines L1–L4 BMD (g/cm²)[e]**								
Year 1 vs baseline	0.8 [0.1-1.6]	0.9 [0.1-1.7]	0.1 [–1.1-1.2]	.99	0.8 [0.1-1.6]	0.9 [0.1-1.7]	0.1 [–1.1-1.2]	.98
Year 3 vs baseline	1.3 [0.6-2.1]	1.4 [0.6-2.2]	0.1 [–1.0-1.2]		1.3 [0.6-2.1]	1.4 [0.6-2.2]	0.1 [–1.0-1.2]	
**Femoral trochanter BMD (g/cm²)[e]**								
Year 1 vs baseline	–0.4 [–1.1-0.4]	0.8 [–0.1-1.7]	1.1 [0.1-2.2]	.07	–0.4 [–1.1-0.4]	0.8 [–0.1-1.7]	1.1 [0.1-2.2]	.06
Year 3 vs baseline	–0.4 [–1.2-0.3]	–0.4 [–1.2-0.4]	0.1 [–1.1-1.1]		–0.4 [–1.2-0.3]	–0.4 [–1.2-0.4]	0.1 [–1.1-1.1]	
**Total bone mineral content (g)**								
Year 1 vs baseline	–11.0 [–23.5-1.5]	–4.4 [–17.0-8.2]	6.6 [–11.2-24.4]	.65	–11.0 [–23.5-1.5]	–4.4 [–17.0-8.2]	6.6 [–11.2-24.4]	.64
Year 3 vs baseline	–12.6 [–25.1 to –0.1]	–14.5 [–27.4 to –1.6]	–1.9 [–19.9-16.0]		–12.6 [–25.1 to –0.1]	–14.5 [–27.4 to –1.6]	–1.9 [–19.9-16.0]	
**Women[d]**								
**Variable**	**Basic model**				**Multivariable-adjusted model**			
**Bone variable**	**Changes in control**	**Changes in intervention**	**Mean difference in changes**	**P-value[f]**	**Changes in control**	**Changes in intervention**	**Mean difference in changes**	**P-value[f]**
**Total femur BMD (g/cm²)[e]**								
Year 1 vs baseline	–0.1 [–0.5-0.5]	–0.8 [–1.4 to –0.3]	–0.8 [–1.6 to –0.1]	.09	–0.1 [–0.5-0.5]	–0.8 [–1.4 to –0.3]	–0.8 [–1.6 to –0.1]	.08
Year 3 vs baseline	–1.5 [–2.1 to –1.0]	–1.6 [–2.2 to –1.0]	–0.1 [–0.9-0.7]		–1.5 [–2.1 to –1.0]	–1.6 [–2.2 to –1.0]	–0.1 [–0.9-0.7]	
**Lumbar spines L1–L4 BMD (g/cm²)[e]**								
Year 1 vs baseline	0.1 [–0.7-0.9]	–0.4 [–1.8-1.1]	–0.1 [–1.3-1.1]	.005	0.1 [–0.7-0.9]	–0.4 [–1.8-1.1]	–0.1 [–1.3-1.1]	.005
Year 3 vs baseline	–1.3 [–2.2 to –0.5]	0.4 [–0.5-0.5]	1.8 [0.6-2.9]		–1.3 [–2.2 to -0.5]	0.4 [–0.5-0.5]	1.8 [0.6-2.9]	
**Femoral trochanter BMD (g/cm²)[e]**								
Year 1 vs baseline	0.1 [–0.6-0.8]	–0.7 [–1.5-0.1]	–0.8 [–1.9-0.2]	.13	0.1 [–0.6-0.8]	–0.7 [–1.5-0.1]	–0.8 [–1.9-0.2]	.12
Year 3 vs baseline	–1.2 [–1.9 to –0.5]	–0.9 [–1.7 to –0.2]	0.3 [–0.8-1.3]		–1.2 [–1.9 to –0.5]	–0.9 [–1.7 to –0.2]	0.3 [–0.8-1.3]	
**Total bone mineral content (g)**								
Year 1 vs baseline	–9.6 [–20.8-1.5]	–22.9 [–34.8 to –13.5]	–13.2 [–29.5-3.1]	.23	–9.6 [–20.8-1.5]	–22.9 [–34.8 to –13.5]	–13.2 [–29.5-3.1]	.23
Year 3 vs baseline	–24.7 [–35.9 to –13.5]	–25.7 [–37.8 to –13.5]	–0.9 [–17.5-15.6]		–24.7 [–35.9 to –13.5]	–25.7 [–37.8 to –13.5]	–0.9 [–17.5-15.6]	

© 2025 Vázquez-Lorente H et al. *JAMA Network Open*.

Abbreviations: BMC, bone mineral content. BMD, bone mineral density. CI, confidence interval. PREDIMED-Plus, Prevención con Dieta Mediterránea-Plus.
[a]Two-level linear mixed models were fitted with random intercepts at cluster family (as couples from the same household were randomized together) and individual participants to assess intervention group effects on changes in BMD variables and total BMC repeatedly measured over time (at each follow-up visit and for the overall follow-up period). An interaction term between the intervention group and time, age (years), sex (men/women), and recruiting center (Navarra, Mallorca, Reus, and León) were included as fixed effects in the basic model. Baseline education level (primary or less, secondary, or college), civil status (single, divorced or separated, married, widower), smoking status (current, former, or never), diabetes prevalence (yes/no), hypertension prevalence (yes/no), hypercholesterolemia prevalence (yes/no), body mass index (kg/m^2), physical activity (METs/min/day), sedentary time (h/day), calcium and/or vitamin D medication/supplementation (yes/no), use of osteoporotic drugs (yes/no), adherence to the energy-restricted Mediterranean diet (0-17 points), and daily energy intake (kcal/day) were additionally included as fixed effects in the multivariable-adjusted models. Sex (men/women) was excluded as a covariate in the analyses stratified for sex. Data are presented as mean [95% CI]. Significance was set at P-values ≤.05.
[b]Overall, n=924; Control group, n=464; Intervention group, n=460.
[c]Men, n=470; Control group, n=233; Intervention group, n=237.
[d]Women, n=454; Control group, n=231; Intervention group, n=223.
[e]Expressed as multiples of 10^{-2} (x10^{-2}).
[f]P-value represents the intervention group effects assessed for the overall follow-up period.

© 2025 Vázquez-Lorente H et al. *JAMA Network Open*.

eTable 4. Effect of Intervention Group on Low BMD Prevalence Over 3 Years in the Overall Population and by Sex[a]

Overall[b]				
**Variable**	**Basic model**		**Multivariable-adjusted model**	
**Bone variable**	**Intervention Group**	**Overall P-value[e]**	**Intervention Group**	**Overall P-value[e]**
**Total femur**				
Year 1 vs baseline	2.1 [0.9-4.8]	.13	2.2 [0.6-7.4]	.13
Year 3 vs baseline	2.1 [0.9-4.9]		3.9 [1.1-14.5]	
**Lumbar spines L1–L4**				
Year 1 vs baseline	0.6 [0.2-1.6]	.55	0.6 [0.2-2.5]	.59
Year 3 vs baseline	1.0 [0.4-2.6]		1.8 [0.4-7.4]	
**Femoral trochanter**				
Year 1 vs baseline	1.2 [0.5-2.5]	.13	0.5 [0.2-1.7]	.13
Year 3 vs baseline	2.1 [1.0-4.6]		2.3 [0.7-7.1]	
**Men[c]**				
**Variable**	**Basic model**		**Multivariable-adjusted model**	
**Bone variable**	**Intervention Group**	**Overall P-value[e]**	**Intervention Group**	**Overall P-value[e]**
**Total femur**				
Year 1 vs baseline	2.2 [0.6-7.4]	.12	2.2 [0.6-7.4]	.12
Year 3 vs baseline	3.9 [1.1-14.5]		3.9 [1.1-14.5]	
**Lumbar spines L1–L4**				
Year 1 vs baseline	0.6 [0.2-2.5]	.41	0.6 [0.2-2.5]	.37
Year 3 vs baseline	1.7 [0.4-6.8]		1.8 [0.4-7.4]	
**Femoral trochanter**				
Year 1 vs baseline	0.6 [0.2-1.8]	.08	0.5 [0.2-1.7]	.08
Year 3 vs baseline	2.3 [0.7-7.1]		2.3 [0.7-7.1]	
**Women[d]**				
**Variable**	**Basic model**		**Multivariable-adjusted model**	
**Bone variable**	**Intervention Group**	**Overall P-value[e]**	**Intervention Group**	**Overall P-value[e]**
**Total femur**				
Year 1 vs baseline	2.1 [0.7-6.6]	.43	2.1 [0.7-6.6]	.43
Year 3 vs baseline	1.3 [0.4-4.0]		1.3 [0.4-4.0]	
**Lumbar spines L1–L4**				
Year 1 vs baseline	0.6 [0.1-2.5]	.68	0.6 [0.1-2.5]	.69
Year 3 vs baseline	0.6 [0.2-2.2]		0.6 [0.2-2.2]	
**Femoral trochanter**				
Year 1 vs baseline	2.2 [0.8-6.3]	.23	2.2 [0.8-6.3]	.23
Year 3 vs baseline	2.1 [0.7-5.9]		2.1 [0.7-5.9]	

© 2025 Vázquez-Lorente H et al. *JAMA Network Open*.

Abbreviations: BMD, bone mineral density. CI, confidence interval. OR, odds ratio.

[a]Two-level logistic mixed models were fitted with random intercepts at cluster family (as couples from the same household were randomized together) and individual participants to assess the intervention group effect on low BMD prevalence. BMD was considered low for all participants with a T-score less than − 1 (osteopenia/osteoporosis) in every BMD measure. An interaction term between the intervention group and time, age (years), sex (men/women), and recruiting center (Navarra, Mallorca, Reus, and León) were included as fixed effects in the basic model. Baseline education level (primary or less, secondary, or college), civil status (single, divorced or separated, married, widower), smoking status (current, former, or never), diabetes prevalence (yes/no), hypertension prevalence (yes/no), hypercholesterolemia prevalence (yes/no), body mass index (kg/m^2) physical activity (METs/min/day), sedentary time (h/day), calcium and/or vitamin D medication/supplementation (yes/no), use of osteoporotic drugs (yes/no), adherence to the energy-restricted Mediterranean diet (0-17 points), and daily energy intake (kcal/day) were additionally included in the multivariable-adjusted model. Sex (men/women) was excluded as a covariate in the analyses stratified for sex. The control group was considered as 1 (reference). Data are presented as OR [95% CI]. Significance was set at P-values ≤.05.

[b]Overall, n=924; Control group, n=464; Intervention group, n=460.

[c]Men, n=470; Control group, n=233; Intervention group, n=237.

[d]Women, n=454; Control group, n=231; Intervention group, n=223.

[e]P-value represents the intervention group effects assessed for the overall follow-up period.

© 2025 Vázquez-Lorente H et al. *JAMA Network Open*.

eTable 5. Effect of the PREDIMED-Plus Intervention on BMD Variables and Total BMC Content Over 3 Years After Multiple Imputation in the Overall Population and by Sex (Intention-to-Treat Analysis)[a]

Overall[b]								
**Variable**	**Basic model**				**Multivariable-adjusted model**			
**Bone variable**	**Changes in control**	**Changes in intervention**	**Mean difference in changes**	**P-value[f]**	**Changes in control**	**Changes in intervention**	**Mean difference in changes**	**P-value[f]**
**Total femur BMD (g/cm²)[e]**								
Year 1 vs baseline	–0.3 [–1.0-0.4]	0.1 [–0.6-0.8]	0.4 [–0.6-1.4]	.17	–0.3 [–1.0-0.4]	0.1 [–0.6-0.8]	0.4 [–0.6-1.4]	.17
Year 3 vs baseline	–1.5 [–2.2 to –0.8]	–0.5 [–1.2-0.2]	0.9 [–0.1-2.0]		–1.5 [–2.2 to –0.8]	–0.5 [–1.2-0.2]	0.9 [–0.1-2.0]	
**Lumbar spines L1–L4 BMD (g/cm²)[e]**								
Year 1 vs baseline	–0.1 [–1.2-1.0]	–0.1 [–1.2-1.1]	0.1 [–1.6-1.6]	.41	–0.1 [–1.2-1.0]	–0.1 [–1.2-1.1]	0.1 [–1.6-1.6]	.41
Year 3 vs baseline	–0.3 [–1.5-0.8]	0.6 [–0.5-1.8]	1.0 [–0.7-2.6]		–0.3 [–1.5-0.8]	0.6 [–0.5-1.8]	1.0 [–0.7-2.6]	
**Femoral trochanter BMD (g/cm²)[e]**								
Year 1 vs baseline	–0.4 [–1.2-0.3]	0.3 [–0.4-1.1]	0.8 [–0.3-1.8]	.06	–0.4 [–1.2-0.3]	0.3 [–0.4-1.1]	0.8 [–0.3-1.8]	.06
Year 3 vs baseline	–1.4 [–2.1 to –0.7]	–0.1 [–0.9-0.6]	1.3 [0.2-2.3]		–1.4 [–2.1 to –0.7]	–0.1 [–0.9-0.6]	1.3 [0.2-2.3]	
**Total bone mineral content (g)**								
Year 1 vs baseline	–17.0 [–38.1–4.1]	0.7 [–20.5-21.9]	17.7 [–12.2-47.6]	.41	–17.0 [–38.1-4.1]	0.7 [–20.5-21.9]	17.7 [–12.2-47.6]	.41
Year 3 vs baseline	–25.7 [–48.8 to –2.5]	–6.7 [–31.0-17.5]	18.9 [–14.6-52.4]		–25.7 [–48.8 to –2.5]	–6.7 [–31.0-17.5]	18.9 [–14.6-52.4]	

Men[c]								
**Variable**	**Basic model**				**Multivariable-adjusted model**			
**Bone variable**	**Changes in control**	**Changes in intervention**	**Mean difference in changes**	**P-value[f]**	**Changes in control**	**Changes in intervention**	**Mean difference in changes**	**P-value[f]**
**Total femur BMD (g/cm²)[e]**								
Year 1 vs baseline	–0.6 [–1.5-0.4]	0.5 [–0.4-1.5]	1.1 [–0.2-2.4]	.22	–0.6 [–1.5-0.4]	0.5 [–0.4-1.5]	1.1 [–0.2-2.4]	.22
Year 3 vs baseline	–1.2 [–2.1 to –0.2]	–0.3 [–1.2-0.7]	0.9 [–0.4-2.2]		–1.2 [–2.1 to –0.2]	–0.3 [–1.2-0.7]	0.9 [–0.4-2.2]	

© 2025 Vázquez-Lorente H et al. *JAMA Network Open*.

**Lumbar spines L1–L4 BMD (g/cm^2)[e]**								
Year 1 vs baseline	0.4 [–1.2-2.0]	–0.3 [–1.9-1.2]	–0.8 [–3.0-1.5]	.80	0.4 [–1.2-2.0]	–0.3 [–1.9-1.2]	–0.8 [–3.0-1.5]	.80
Year 3 vs baseline	1.2 [–0.4-2.8]	0.9 [–0.7-2.4]	–0.3 [–2.5-1.9]		1.2 [–0.4-2.8]	0.9 [–0.7-2.4]	–0.3 [–2.5-1.9]	
**Femoral trochanter BMD (g/cm^2)[e]**								
Year 1 vs baseline	–0.6 [–1.6-0.4]	0.9 [–0.1-1.9]	1.5 [0.1-2.9]	.08	–0.6 [–1.6-0.4]	0.9 [–0.1-1.9]	1.5 [0.1-2.9]	.08
Year 3 vs baseline	–0.8 [–1.8-0.1]	0.3 [–0.7-1.3]	1.1[–0.2-2.5]		–0.8 [–1.8-0.1]	0.3 [–0.7-1.3]	1.1[–0.2-2.5]	
**Total bone mineral content (g)**								
Year 1 vs baseline	–23.4 [–45.5 to –1.4]	5.7 [–16.1-27.6]	29.1 [–1.9-60.2]	.16	–23.4 [–45.5 to –1.4]	5.7 [–16.1-27.6]	29.1 [–1.9-60.2]	.16
Year 3 vs baseline	–15.0 [–39.1–9.1]	–8.1 [–32.9-16.7]	6.9 [–27.7-41.5]		–15.0 [–39.1-9.1]	–8.1 [–32.9-16.7]	6.9 [–27.7-41.5]	
**Women[d]**								
**Variable**	**Basic model**				**Multivariable-adjusted model**			
**Bone variable**	**Changes in control**	**Changes in intervention**	**Mean difference in changes**	**P-value[f]**	**Changes in control**	**Changes in intervention**	**Mean difference in changes**	**P-value[f]**
**Total femur BMD (g/cm^2)[e]**								
Year 1 vs baseline	–0.3 [–1.2-0.6]	–0.9 [–1.8-0.1]	–0.6 [–1.9-0.7]	.05	–0.3 [–1.2-0.6]	–0.9 [–1.8-0.1]	–0.6 [–1.9-0.7]	.05
Year 3 vs baseline	–2.1 [–3.0 to –1.1]	–1.1 [–2.0 to –0.1]	1.0 [–0.3-2.3]		–2.1 [–3.0 to –1.1]	–1.1 [–2.0 to –0.1]	1.0 [–0.3-2.3]	
**Lumbar spines L1–L4 BMD (g/cm^2)[e]**								
Year 1 vs baseline	–0.5 [–1.8-0.9]	–0.2 [–1.6-1.2]	0.2 [–1.7-2.2]	.05	–0.5 [–1.8-0.9]	–0.2 [–1.6-1.2]	0.2 [–1.7-2.2]	.05
Year 3 vs baseline	–1.8 [–3.2 to –0.4]	0.5 [–0.9-1.9]	2.2 [0.3-4.2]		–1.8 [–3.2 to –0.4]	0.5 [–0.9-1.9]	2.2 [0.3-4.2]	
**Femoral trochanter BMD (g/cm^2)[e]**								
Year 1 vs baseline	–0.3 [–1.2-0.6]	–0.8 [–1.7-0.2]	–0.5 [–1.8-0.9]	.01	–0.3 [–1.2-0.6]	–0.8 [–1.7-0.2]	–0.5 [–1.8-0.9]	.01
Year 3 vs baseline	–2.1 [–3.0 to –1.2]	–0.6 [1.6-0.3]	1.4 [0.1-2.8]		–2.1 [–3.0 to –1.2]	–0.6 [1.6-0.3]	1.4 [0.1-2.8]	
**Total bone mineral content (g)**								
Year 1 vs baseline	–14.3 [–31.7-3.2]	–23.1 [–40.9 to –5.3]	–8.8 [–33.7-16.1]	.45	–14.3 [–31.7-3.2]	–23.1 [–40.9 to –5.3]	–8.8 [–33.7-16.1]	.44
Year 3 vs baseline	–29.1 [–48.4 to –9.9]	–20.0 [–40.5-0.6]	9.2 [–19.0-37.4]		–29.2 [–48.5 to –10.0]	–19.6 [–40.2-1.0]	9.6 [–18.6-37.8]	

© 2025 Vázquez-Lorente H et al. *JAMA Network Open*.

Abbreviations: BMC, bone mineral content. BMD, bone mineral density. CI, confidence interval. PREDIMED-Plus, Prevención con Dieta Mediterránea-Plus.

[a]Two-level linear mixed models were fitted with random intercepts at cluster family (as couples from the same household were randomized together) and individual participants to assess intervention group effects on changes in BMD variables and total BMC measured repeatedly over time (at each follow-up visit and for the overall follow-up period). An interaction term between the intervention group and time, age (years), sex (men/women), and recruiting center (Navarra, Mallorca, Reus, and León) were included as fixed effects in the basic model. Baseline education level (primary or less, secondary, or college), civil status (single, divorced or separated, married, widower), smoking status (current, former, or never), diabetes prevalence (yes/no), hypertension prevalence (yes/no), hypercholesterolemia prevalence (yes/no), body mass index (kg/m^2) physical activity (METs/min/day), sedentary time (h/day), calcium and/or vitamin D medication/supplementation (yes/no), use of osteoporotic drugs (yes/no), adherence to the energy-restricted Mediterranean diet (0-17 points), and daily energy intake (kcal/day) were additionally included as fixed effects in the multivariable-adjusted models. Sex (men/women) was included as a covariate for those analyses performed in the overall population. All missing variables for BMD variables and total BMC over one and three years of follow-up were imputed. Sex (men/women) was excluded as a covariate in the analyses stratified for sex. Data are presented as mean [95% CI]. Significance was set at P-values ≤.05.

[b]Overall, n=924; Control group, n=464; Intervention group, n=460.

[c]Men, n=470; Control group, n=233; Intervention group, n=237.

[d]Women, n=454; Control group, n=231; Intervention group, n=223.

[e]Expressed as multiples of 10^{-2} ($x10^{-2}$).

[f]P-value represents the intervention group effects assessed for the overall follow-up period.

© 2025 Vázquez-Lorente H et al. *JAMA Network Open*.

eTable 6. Effect of Intervention Group on Low BMD Prevalence Over 3 Years After Multiple Imputation in the Overall Population and by Sex (Intention-to-Treat Analysis)[a]

Overall[b]				
Variable	Basic model		Multivariable-adjusted model	
Bone variable	Intervention Group	Overall P-value[e]	Intervention Group	Overall P-value[e]
**Total femur**				
Year 1 vs baseline	1.3 [0.6-2.6]	.56	1.3 [0.6-2.6]	.56
Year 3 vs baseline	0.9 [0.5-1.8]		0.9 [0.5-1.8]	
**Lumbar spines L1–L4**				
Year 1 vs baseline	0.6 [0.3-1.2]	.27	0.6 [0.3-1.2]	.27
Year 3 vs baseline	0.7 [0.3-1.3]		0.7 [0.3-1.3]	
**Femoral trochanter**				
Year 1 vs baseline	0.9 [0.5-1.8]	.79	0.9 [0.5-1.8]	.79
Year 3 vs baseline	1.2 [0.6-2.3]		1.2 [0.6-2.3]	
**Men[c]**				
Variable	Basic model		Multivariable-adjusted model	
Bone variable	Intervention Group	Overall P-value[e]	Intervention Group	Overall P-value[e]
**Total femur**				
Year 1 vs baseline	1.6 [0.5-4.9]	.72	1.5 [0.5-4.8]	.72
Year 3 vs baseline	1.4 [0.5-4.4]		1.4 [0.5-4.3]	
**Lumbar spines L1–L4**				
Year 1 vs baseline	0.7 [0.2-2.4]	.57	0.7 [0.2-2.3]	.56
Year 3 vs baseline	1.3 [0.4-4.3]		1.4 [0.4-4.3]	
**Femoral trochanter**				
Year 1 vs baseline	0.5 [0.2-1.8]	.35	0.5 [0.2-1.8]	.35
Year 3 vs baseline	1.1 [0.4-3.0]		1.1 [0.4-2.9]	
**Women[d]**				
Variable	Basic model		Multivariable-adjusted model	
Bone variable	Intervention Group	Overall P-value[e]	Intervention Group	Overall P-value[e]
**Total femur**				
Year 1 vs baseline	1.2 [0.5-3.0]	.42	1.2 [0.5-3.0]	.42
Year 3 vs baseline	0.6 [0.3-1.6]		0.6 [0.3-1.6]	
**Lumbar spines L1–L4**				
Year 1 vs baseline	0.5 [0.2-1.2]	.11	0.5 [0.2-1.2]	.12
Year 3 vs baseline	0.4 [0.2-1.0]		0.4 [0.2-1.0]	
**Femoral trochanter**				
Year 1 vs baseline	1.5 [0.6-3.9]	.70	1.5 [0.6-3.9]	.70
Year 3 vs baseline	1.3 [0.5-3.3]		1.3 [0.5-3.3]	

© 2025 Vázquez-Lorente H et al. *JAMA Network Open*.

Abbreviations: BMD, bone mineral density. CI, confidence interval. OR, odds ratio.

[a]Two-level logistic mixed models were fitted with random intercepts at cluster family (as couples from the same household were randomized together) and individual participants to assess the intervention group effect on low BMD prevalence. BMD was considered low for all participants with a T-score less than − 1 (osteopenia/osteoporosis) in every BMD measure. An interaction term between the intervention group and time, age (years), sex (men/women), and recruiting center (Navarra, Mallorca, Reus, and León) were included as fixed effects in the basic model. Baseline education level (primary or less, secondary, or college), civil status (single, divorced or separated, married, widower), smoking status (current, former, or never), diabetes prevalence (yes/no), hypertension prevalence (yes/no), hypercholesterolemia prevalence (yes/no), body mass index (kg/m^2) physical activity (METs/min/day), sedentary time (h/day), calcium and/or vitamin D medication/supplementation (yes/no), use of osteoporotic drugs (yes/no), adherence to the energy-restricted Mediterranean diet (0-17 points), and daily energy intake (kcal/day) were additionally included in the multivariable-adjusted model. All missing variables for T-scores over one and three years of follow-up were imputed. Sex (men/women) was excluded as a covariate in the analyses stratified for sex. The control group was considered as 1 (reference). Data are presented as OR [95% CI]. Significance was set at P-values ≤.05.

[b]Overall, n=924; Control group, n=464; Intervention group, n=460.

[c]Men, n=470; Control group, n=233; Intervention group, n=237.

[d]Women, n=454; Control group, n=231; Intervention group, n=223.

[e]P-value represents the intervention group effects assessed for the overall follow-up period.

© 2025 Vázquez-Lorente H et al. *JAMA Network Open*.

eTable 7. Effect of the PREDIMED-Plus Intervention on BMD Variables and Total BMC Content Over 3 Years After Excluding Participants Lost to Follow-Up in the Overall Population and by Sex (Completers Case Analysis)[a]

Overall[b]								
**Variable**	**Basic model**				**Multivariable-adjusted model**			
**Bone variable**	**Changes in control**	**Changes in intervention**	**Mean difference in changes**	**P-value[f]**	**Changes in control**	**Changes in intervention**	**Mean difference in changes**	**P-value[f]**
**Total femur BMD (g/cm²)[e]**								
Year 1 vs baseline	–0.4 [–0.8-0.1]	–0.3 [–0.8-0.2]	0.1 [–0.6-0.8]	.74	–0.4 [–0.8-0.1]	–0.3 [–0.8-0.2]	0.1 [–0.6-0.8]	.74
Year 3 vs baseline	–1.2 [–1.6 to –0.7]	–1.4 [–1.9 to –0.9]	–0.2 [–0.9-0.5]		–1.2 [–1.6 to –0.7]	–1.4 [–1.9 to –0.9]	–0.2 [–0.9-0.5]	
**Lumbar spines L1–L4 BMD (g/cm²)[e]**								
Year 1 vs baseline	0.7 [–0.1-1.4]	0.5 [–0.3-1.2]	–0.2 [–1.2-0.8]	.19	0.7 [–0.1-1.4]	0.5 [–0.3-1.2]	–0.2 [–1.2-0.8]	.19
Year 3 vs baseline	0.1 [–0.6-0.8]	0.8 [0.1-1.5]	0.7 [–0.3-1.7]		0.1 [–0.6-0.8]	0.8 [0.1-1.5]	0.7 [–0.3-1.7]	
**Femoral trochanter BMD (g/cm²)[e]**								
Year 1 vs baseline	–0.3 [–0.9-0.2]	0.1 [–0.5-0.7]	0.4 [–0.4-1.2]	.62	–0.3 [–0.9-0.2]	0.1 [–0.5-0.7]	0.4 [–0.4-1.2]	.62
Year 3 vs baseline	–1.0 [–1.6 to –0.5]	–0.8 [–1.4 to –0.2]	0.2 [–0.6-1.0]		–1.0 [–1.6 to –0.5]	–0.8 [–1.4 to –0.2]	0.2 [–0.6-1.0]	
**Total bone mineral content (g)**								
Year 1 vs baseline	–11.3 [–21.5 to –1.1]	–13.5 [–24.4 to –2.5]	2.1 [–17.1-12.8]	.96	–11.3 [–21.5 to –1.1]	–13.5 [–24.4 to –2.5]	2.1 [–17.1-12.8]	.96
Year 3 vs baseline	–22.7 [–32.9 to –12.5]	–23.0 [–34.0-12.1]	–0.3 [–15.2-14.7]		–22.7 [–32.9 to –12.5]	–23.0 [–34.0-12.1]	–0.3 [–15.2-14.7]	
**Men[c]**								
**Variable**	**Basic model**				**Multivariable-adjusted model**			
**Bone variable**	**Changes in control**	**Changes in intervention**	**Mean difference in changes**	**P-value[f]**	**Changes in control**	**Changes in intervention**	**Mean difference in changes**	**P-value[f]**
**Total femur BMD (g/cm²)[e]**								
Year 1 vs baseline	–0.5 [–1.1-0.1]	0.1 [–0.6-0.7]	0.6 [–0.3-1.4]	.06	–0.5 [–1.1-0.1]	0.1 [–0.6-0.7]	0.6 [–0.3-1.4]	.06
Year 3 vs baseline	–0.8 [–1.4 to –0.2]	–1.3 [–1.9 to –0.7]	–0.5 [–1.4–0.4]		–0.8 [–1.4 to –0.2]	–1.3 [–1.9 to –0.7]	–0.5 [–1.4-0.4]	

© 2025 Vázquez-Lorente H et al. *JAMA Network Open*.

**Lumbar spines L1–L4 BMD (g/cm²)[e]**								
Year 1 vs baseline	1.1 [0.2-2.0]	1.0 [0.1-2.0]	–0.1 [–1.4-1.2]	.91	1.1 [0.2-2.0]	1.0 [0.1-2.0]	–0.1 [–1.4-1.2]	.91
Year 3 vs baseline	1.6 [0.7-2.5]	1.4 [0.4-2.3]	–0.3 [–1.6-1.0]		1.6 [0.7-2.5]	1.4 [0.4-2.3]	–0.3 [–1.6-1.0]	
**Femoral trochanter BMD (g/cm²)[e]**								
Year 1 vs baseline	–0.3 [–1.1-0.5]	0.6 [–0.2-1.4]	0.9 [–0.2-2.0]	.13	–0.3 [–1.1-0.5]	0.6 [–0.2-1.4]	0.9 [–0.2-2.0]	.13
Year 3 vs baseline	–0.5 [–1.3-0.3]	–0.6 [–1.4-0.2]	–0.1[–1.2-1.0]		–0.5 [–1.3-0.3]	–0.6 [–1.4-0.2]	–0.1[–1.2-1.0]	
**Total bone mineral content (g)**								
Year 1 vs baseline	–17.3 [–32.4 to –2.1]	6.6 [–22.1-9.0]	10.7 [–11.0-32.4]	.62	–23.4 [–45.5 to –1.4]	5.7 [–16.1-27.6]	29.1 [–1.9-60.2]	.16
Year 3 vs baseline	–20.5 [–35.7 to –5.4]	–16.0 [–31.5 to –0.41]	4.6 [–17.1-26.3]		–15.0 [–39.1-9.1]	–8.1 [–32.9-16.7]	6.9 [–27.7-41.5]	
**Women[d]**								
**Variable**	**Basic model**				**Multivariable-adjusted model**			
**Bone variable**	**Changes in control**	**Changes in intervention**	**Mean difference in changes**	**P-value[f]**	**Changes in control**	**Changes in intervention**	**Mean difference in changes**	**P-value[f]**
**Total femur BMD (g/cm²)[e]**								
Year 1 vs baseline	–0.2 [–0.9-0.5]	–0.7 [–1.5-0.1]	–0.5 [–1.6-0.6]	.49	–0.2 [–0.9-0.5]	–0.7 [–1.5-0.1]	–0.5 [–1.6-0.6]	.49
Year 3 vs baseline	–1.6 [–2.3 to –0.8]	–1.4 [–2.3 to –0.6]	0.1 [–1.0-1.2]		–1.6 [–2.3 to –0.8]	–1.4 [–2.3 to –0.6]	0.1 [–1.0-1.2]	
**Lumbar spines L1–L4 BMD (g/cm²)[e]**								
Year 1 vs baseline	0.2 [–0.8-1.3]	–0.2 [–1.4-0.9]	0.2 [–1.7-2.2]	.03	0.2 [–0.8-1.3]	–0.2 [–1.4-0.9]	0.2 [–1.7-2.2]	.03
Year 3 vs baseline	–1.5 [–2.5 to –0.5]	0.1 [–1.1-1.2]	1.6 [0.1-3.1]		–1.5 [–2.5 to –0.5]	0.1 [–1.1-1.2]	1.6 [0.1-3.1]	
**Femoral trochanter BMD (g/cm²)[e]**								
Year 1 vs baseline	–0.4 [–1.2-0.4]	–0.6 [–1.5-0.3]	–0.2 [–1.4-1.0]	.49	–0.4 [–1.2-0.4]	–0.6 [–1.5-0.3]	–0.2 [–1.4-1.0]	.49
Year 3 vs baseline	–1.6 [–2.4 to –0.8]	–1.1 [–2.0-0.2]	0.5 [–0.7-1.7]		–1.6 [–2.4 to –0.8]	–1.1 [–2.0-0.2]	0.5 [–0.7-1.7]	
**Total bone mineral content (g)**								
Year 1 vs baseline	–5.2 [–31.7-3.2]	–18.7 [–37.0 to –6.7]	–16.6 [–36.9-3.7]	.27	–5.2 [–31.7-3.2]	–18.7 [–37.0 to –6.7]	–16.6 [–36.9-3.7]	.27
Year 3 vs baseline	–25.0 [–38.5 to –11.5]	–31.7 [–46.8 to –16.5]	–6.7 [–26.9-13.6]		–25.0 [–38.5 to –11.5]	–31.7 [–46.8 to –16.5]	–6.7 [–26.9-13.6]	

© 2025 Vázquez-Lorente H et al. *JAMA Network Open*.

Abbreviations: BMC, bone mineral content. BMD, bone mineral density. CI, confidence interval. PREDIMED-Plus, Prevención con Dieta Mediterránea-Plus.

[a]Two-level linear mixed models were fitted with random intercepts at cluster family (as couples from the same household were randomized together) and individual participants to assess intervention group effects on changes in BMD variables and total BMC repeatedly measured over time (at each follow-up visit and for the overall follow-up period). An interaction term between the intervention group and time, age (years), sex (men/women), and recruiting center (Navarra, Mallorca, Reus, and León) were included as fixed effects in the basic model. Baseline education level (primary or less, secondary, or college), civil status (single, divorced or separated, married, widower), smoking status (current, former, or never), diabetes prevalence (yes/no), hypertension prevalence (yes/no), hypercholesterolemia prevalence (yes/no), body mass index (kg/m^2) physical activity (METs/min/day), sedentary time (h/day), calcium and/or vitamin D medication/supplementation (yes/no), use of osteoporotic drugs (yes/no), adherence to the energy-restricted Mediterranean diet (0-17 points), and daily energy intake (kcal/day) were additionally included as fixed effects in the multivariable-adjusted models. Sex (men/women) was included as a covariate for those analyses performed in the overall population. All missing variables for T-scores over one and three years of follow-up were eliminated. Sex (men/women) was excluded as a covariate in the analyses stratified for sex. Data are presented as mean [95% CI]. Significance was set at P-values ≤.05.

[b]Overall, n=438; Control group, n=234; Intervention group, n=204.

[c]Men, n=230; Control group, n=118; Intervention group, n=112.

[d]Women, n=208; Control group, n=116; Intervention group, n=92.

[e]Expressed as multiples of 10^{-2} (x10^{-2}).

[f]P-value represents the intervention group effects assessed for the overall follow-up period.

© 2025 Vázquez-Lorente H et al. *JAMA Network Open*.

eTable 8. Effect of Intervention Group on Low BMD Prevalence Over 3 Years After Excluding Participants Lost to Follow-Up in the Overall Population and by Sex (Completers Case Analysis)[a]

Variable	Basic model		Multivariable-adjusted model	
**Overall[b]**				
**Bone variable**	**Intervention Group**	**Overall P-value[e]**	**Intervention Group**	**Overall P-value[e]**
**Total femur**				
Year 1 vs baseline	2.2 [0.6-8.3]	.45	2.2 [0.6-8.3]	.45
Year 3 vs baseline	1.2 [0.3-4.4]		1.2 [0.3-4.3]	
**Lumbar spines L1–L4**				
Year 1 vs baseline	0.7 [0.2-2.9]	.70	0.7 [0.2-2.9]	.69
Year 3 vs baseline	1.3 [0.3-5.1]		1.3 [0.4-5.1]	
**Femoral trochanter**				
Year 1 vs baseline	1.1 [0.4-3.1]	.54	1.1 [0.4-3.1]	.54
Year 3 vs baseline	1.7 [0.6-5.0]		1.7 [0.6-5.0]	
**Men[c]**				
**Variable**	**Basic model**		**Multivariable-adjusted model**	
**Bone variable**	**Intervention Group**	**Overall P-value[e]**	**Intervention Group**	**Overall P-value[e]**
**Total femur**				
Year 1 vs baseline	2.4 [0.3-23.5]	.65	2.0 [0.2-18.1]	.77
Year 3 vs baseline	3.0 [0.2-36.5]		2.0 [0.2-17.6]	
**Lumbar spines L1–L4**				
Year 1 vs baseline	0.6 [0.1-4.3]	.35	0.6 [0.1-4.1]	.29
Year 3 vs baseline	2.6 [0.4-18.3]		2.7 [0.4-19.0]	
**Femoral trochanter**				
Year 1 vs baseline	0.4 [0.1-2.2]	.32	0.4 [0.1-2.3]	.34
Year 3 vs baseline	1.4 [0.3-7.1]		1.5 [0.3-7.1]	
**Women[d]**				
**Variable**	**Basic model**		**Multivariable-adjusted model**	
**Bone variable**	**Intervention Group**	**Overall P-value[e]**	**Intervention Group**	**Overall P-value[e]**
**Total femur**				
Year 1 vs baseline	2.1 [0.4-9.7]	.42	2.0 [0.4-9.3]	.51
Year 3 vs baseline	0.9 [0.2-4.4]		0.9 [0.2-4.0]	
**Lumbar spines L1–L4**				
Year 1 vs baseline	0.9 [0.1-5.8]	.11	0.9 [0.1-7.3]	.88
Year 3 vs baseline	0.7 [0.1-4.3]		0.6 [0.1-4.7]	
**Femoral trochanter**				
Year 1 vs baseline	2.1 [0.5-8.5]	.53	2.1 [0.5-8.5]	.54
Year 3 vs baseline	1.9 [0.5-7.8]		1.9 [0.5-7.7]	

© 2025 Vázquez-Lorente H et al. *JAMA Network Open*.

Abbreviations: BMD, bone mineral density. CI, confidence interval. OR, odds ratio.
[a]Two-level logistic mixed models were fitted with random intercepts at the cluster family (as couples from the same household were randomized together) and individual participants to assess the intervention group effect on low BMD prevalence. BMD was considered low for all participants with a T-score less than − 1 (osteopenia/osteoporosis) in every BMD measure. An interaction term between the intervention group and time, age (years), sex (men/women), and recruiting center (Navarra, Mallorca, Reus, and León) were included as fixed effects in the basic model. Baseline education level (primary or less, secondary, or college), civil status (single, divorced or separated, married, widower), smoking status (current, former, or never), diabetes prevalence (yes/no), hypertension prevalence (yes/no), hypercholesterolemia prevalence (yes/no), body mass index (kg/m^2) physical activity (METs/min/day), sedentary time (h/day), calcium and/or vitamin D medication/supplementation (yes/no), use of osteoporotic drugs (yes/no), adherence to the energy-restricted Mediterranean diet (0-17 points), and daily energy intake (kcal/day) were additionally included in the multivariable-adjusted model. All missing variables for T-scores over one and three years of follow-up were eliminated. Sex (men/women) was excluded as a covariate in the analyses stratified for sex. The control group was considered as 1 (reference). Data are presented as OR [95% CI]. Significance was set at P-values ≤.05.
[b]Overall, n=438; Control group, n=234; Intervention group, n=204.
[c]Men, n=230; Control group, n=118; Intervention group, n=112.
[d]Women, n=208; Control group, n=116; Intervention group, n=92.
[e]P-value for differences intergroups.
[f]P-value represents the intervention group effects assessed for the overall follow-up period.

© 2025 Vázquez-Lorente H et al. *JAMA Network Open*.

eTable 9. Effect of the PREDIMED-Plus Intervention on BMD Variables and Total BMC Content Over 3 Years After Excluding Participants Taking Calcium or Vitamin D Medication or Supplementation (or Both) in the Overall Population and by Sex[a]

Overall[b]								
**Variable**	**Basic model**				**Multivariable-adjusted model**			
**Bone variable**	**Changes in control**	**Changes in intervention**	**Mean difference in changes**	**P-value[f]**	**Changes in control**	**Changes in intervention**	**Mean difference in changes**	**P-value[f]**
**Total femur BMD (g/cm²)[e]**								
Year 1 vs baseline	–0.1 [–0.5-0.4]	–0.2 [–0.7-0.3]	–0.2 [–0.8-0.5]	.10	–0.1 [–0.5-0.4]	–0.2 [–0.7-0.3]	–0.2 [–0.8-0.5]	.10
Year 3 vs baseline	–0.7 [–1.2 to –0.3]	–1.4 [–1.9 to –0.9]	–0.7 [–1.4 to –0.1]		–0.7 [–1.2 to –0.3]	–1.4 [–1.9 to –0.9]	–0.7 [–1.4 to –0.1]	
**Lumbar spines L1–L4 BMD (g/cm²)[e]**								
Year 1 vs baseline	0.6 [–0.1-1.2]	0.6 [–0.1-1.3]	0.1 [–0.9-1.0]	.15	0.6 [–0.1-1.2]	0.6 [–0.1-1.3]	0.1 [–0.9-1.0]	.15
Year 3 vs baseline	0.1 [–0.5-0.8]	1.0 [0.3-1.6]	0.8 [–0.1-1.8]		0.1 [–0.5-0.8]	1.0 [0.3-1.6]	0.8 [–0.1-1.8]	
**Femoral trochanter BMD (g/cm²)[e]**								
Year 1 vs baseline	–0.1 [–0.7-0.5]	0.2 [–0.4-0.8]	0.3 [–0.6-1.2]	.79	–0.1 [–0.7-0.5]	0.2 [–0.4-0.8]	0.3 [–0.6-1.2]	.79
Year 3 vs baseline	–0.5 [–1.1-0.1]	–0.5 [–1.1-0.1]	0.1 [–0.9-0.9]		–0.6 [–1.1-0.1]	–0.5 [–1.1-0.1]	0.1 [–0.8-0.9]	
**Total bone mineral content (g)**								
Year 1 vs baseline	–6.4 [–15.9-3.0]	–14.7 [–24.4-4.9]	–8.2 [–21.8-5.4]	.46	–6.4 [–15.9-3.0]	–14.6 [–24.4-4.9]	–8.2 [–21.8-5.4]	.46
Year 3 vs baseline	–16.3 [–25.7 to –6.8]	–17.4 [–27.4 to –7.5]	–1.1 [–14.9-12.6]		–16.3 [–25.7 to –6.8]	–17.4 [–27.4 to –7.4]	–1.1 [–14.9-12.6]	
**Men[c]**								
**Variable**	**Basic model**				**Multivariable-adjusted model**			
**Bone variable**	**Changes in control**	**Changes in intervention**	**Mean difference in changes**	**P-value[f]**	**Changes in control**	**Changes in intervention**	**Mean difference in changes**	**P-value[f]**
**Total femur BMD (g/cm²)[e]**								
Year 1 vs baseline	–0.1 [–0.7-0.4]	0.1 [–0.5-0.7]	0.2 [–0.6-1.1]	.06	–0.1 [–0.7-0.4]	0.1 [–0.5-0.7]	0.2 [–0.6-1.1]	.06
Year 3 vs baseline	–0.5 [–1.1-0.1]	–1.4 [–2.0 to –0.7]	–0.8 [–1.7-0.1]		–0.5 [–1.1-0.1]	–1.4 [–2.0 to –0.7]	–0.8 [–1.7-0.1]	
**Lumbar spines L1–L4 BMD (g/cm²)[e]**								
Year 1 vs baseline	0.9 [0.1-1.7]	1.1 [0.2-1.9]	0.2 [–1.0-1.3]	.92	0.9 [0.1-1.7]	1.1 [0.2-1.9]	0.2 [–1.0-1.3]	.91

© 2025 Vázquez-Lorente H et al. *JAMA Network Open*.

Year 3 vs baseline	1.4 [0.6-2.2]	1.3 [0.5-2.2]	–0.1 [–1.3-1.1]		1.4 [0.6-2.2]	1.3 [0.5-2.2]	–0.1 [–1.3-1.1]	
**Femoral trochanter BMD (g/cm^2)[e]**								
Year 1 vs baseline	–0.4 [–1.2-0.4]	0.7 [–0.1-1.5]	1.1 [0.1-2.3]	.09	–0.4 [–1.2-0.4]	0.7 [–0.1-1.5]	1.1 [0.1-2.3]	.09
Year 3 vs baseline	–0.5 [–1.3-0.3]	–0.4 [–1.3-0.4]	0.1 [–1.1-1.2]		–0.5 [–1.3-0.3]	–0.4 [–1.3-0.4]	0.1 [–1.1-1.2]	
**Total bone mineral content (g)**								
Year 1 vs baseline	–8.5 [–21.5-4.6]	–5.0 [–18.3-8.4]	3.5 [–15.2-22.2]	.90	–8.5 [–21.5-4.6]	–5.0 [–18.3-8.4]	3.5 [–15.2-22.2]	.90
Year 3 vs baseline	–11.9 [–24.9-1.1]	–12.7 [–26.4-1.0]	–0.8 [–19.7-18.1]		–11.9 [–24.9-1.1]	–12.7 [–26.4-1.0]	–0.8 [–19.7-18.1]	
				**Women[d]**				
**Variable**	**Basic model**				**Multivariable-adjusted model**			
**Bone variable**	**Changes in control**	**Changes in intervention**	**Mean difference in changes**	**P-value[f]**	**Changes in control**	**Changes in intervention**	**Mean difference in changes**	**P-value[f]**
**Total femur BMD (g/cm^2)[e]**								
Year 1 vs baseline	0.1 [–0.5-0.8]	–0.7 [1.3-0.4]	–0.8 [–1.7-0.2]	.26	0.1 [–0.5-0.8]	–0.6 [1.3-0.4]	–0.8 [–1.7-0.2]	.27
Year 3 vs baseline	–1.0 [–1.7 to –0.4]	–1.5 [–2.2 to –0.8]	–0.5 [–1.5-0.5]		–1.0 [–1.7 to –0.4]	–1.5 [–2.2 to –0.8]	–0.5 [–1.5-0.5]	
**Lumbar spines L1–L4 BMD (g/cm^2)[e]**								
Year 1 vs baseline	0.2 [–0.7-1.2]	0.1 [–1.0-1.1]	–0.2 [–1.6-1.2]	.006	0.2 [–0.7-1.2]	0.1 [–1.0-1.1]	–0.2 [–1.6-1.2]	.006
Year 3 vs baseline	–1.5 [–2.4 to –0.5]	0.5 [–0.5-1.5]	2.0 [0.6-3.4]		–1.5 [–2.4 to –0.5]	0.5 [–0.5-1.5]	2.0 [0.6-3.4]	
**Femoral trochanter BMD (g/cm^2)[e]**								
Year 1 vs baseline	0.4 [–0.5-1.4]	–0.6 [–1.5-0.4]	–1.0 [–2.3-0.4]	.27	0.4 [–0.5-1.4]	–0.5 [–1.5-0.4]	–1.0 [–2.3-0.4]	.27
Year 3 vs baseline	–0.7 [–1.6-0.3]	–0.6 [–1.6-0.3]	0.1 [–1.3-1.4]		–0.7 [–1.6-0.2]	–0.6 [–1.6-0.3]	0.1 [–1.3-1.4]	
**Total bone mineral content (g)**								
Year 1 vs baseline	–3.4 [–16.8-9.9]	–28.9 [–42.7 to –15.1]	–25.5 [–44.6 to –6.3]	.02	–3.4 [–16.8-9.9]	–28.9 [–42.7 to –15.1]	–25.5 [–44.6 to –6.3]	.02
Year 3 vs baseline	–22.7 [–36.0 to –9.4]	–24.5 [–38.6 to –10.4]	–1.8 [–21.1 to –17.6]		–22.7 [–36.0 to –9.4]	–24.5 [–38.6 to –10.4]	–1.8 [–21.1 to –17.6]	

Abbreviations: BMC, bone mineral content. BMD, bone mineral density. CI, confidence interval. PREDIMED-Plus, Prevención con Dieta Mediterránea-Plus.

[a]Two-level linear mixed models were fitted with random intercepts at cluster family (as couples from the same household were randomized together) and individual participants to assess intervention group effects on changes in BMD variables and total BMC measured repeatedly over time (at each follow-up visit and for the overall follow-up period). An interaction term between the intervention

© 2025 Vázquez-Lorente H et al. *JAMA Network Open*.

group and time, age (years), sex (men/women), and recruiting center (Navarra, Mallorca, Reus, and León) were included as fixed effects in the basic model. Baseline education level (primary or less, secondary, or college), civil status (single, divorced or separated, married, widower), smoking status (current, former, or never), diabetes prevalence (yes/no), hypertension prevalence (yes/no), hypercholesterolemia prevalence (yes/no), body mass index (kg/m^2) physical activity (METs/min/day), sedentary time (h/day), use of osteoporotic drugs (yes/no), adherence to the energy-restricted Mediterranean diet (0-17 points), and daily energy intake (kcal/day) were additionally included as fixed effects in the multivariable-adjusted models. Those participants taking calcium and/or vitamin D medication/supplementation at all time points were excluded. Sex (men/women) was excluded as a covariate in the analyses stratified for sex. Data are presented as mean [95% CI]. Significance was set at P-values ≤.05.
[b]Overall, n=736; Control group, n=369; Intervention group, n=367.
[c]Men, n=431; Control group, n=217; Intervention group, n=214.
[d]Women, n=305; Control group, n=152; Intervention group, n=153.
[e]Expressed as multiples of 10^{-2} (x10^{-2}).
[f]P-value represents the intervention group effects assessed for the overall follow-up period.

© 2025 Vázquez-Lorente H et al. *JAMA Network Open*.

eTable 10. Effect of Intervention Group on Low BMD Prevalence Over 3 Years After Excluding Participants Taking Calcium or Vitamin D Medication or Supplementation (or Both) in the Overall Population and by Sex[a]

Variable	Basic model		Multivariable-adjusted model	
**Overall[b]**				
**Bone variable**	**Intervention Group**	**Overall P-value[e]**	**Intervention Group**	**Overall P-value[e]**
**Total femur**				
Year 1 vs baseline	1.8 [0.7-4.7]	.13	1.8 [0.7-4.7]	.13
Year 3 vs baseline	2.7 [1.0-7.3]		2.7 [1.0-7.3]	
**Lumbar spines L1–L4**				
Year 1 vs baseline	0.7 [0.2-2.1]	.74	0.7 [0.2-2.1]	.76
Year 3 vs baseline	1.1 [0.4-3.1]		1.1 [0.4-3.1]	
**Femoral trochanter**				
Year 1 vs baseline	1.0 [0.4-2.8]	.67	1.0 [0.4-2.7]	.66
Year 3 vs baseline	1.5 [0.6-4.0]		1.5 [0.6-3.9]	
**Men[c]**				
**Variable**	**Basic model**		**Multivariable-adjusted model**	
**Bone variable**	**Intervention Group**	**Overall P-value[e]**	**Intervention Group**	**Overall P-value[e]**
**Total femur**				
Year 1 vs baseline	2.3 [0.6-8.9]	.07	2.3 [0.6-8.9]	.07
Year 3 vs baseline	5.1 [1.3-20.7]		5.2 [1.3-20.7]	
**Lumbar spines L1–L4**				
Year 1 vs baseline	0.6 [0.1-2.5]	.36	0.6 [0.1-2.5]	.36
Year 3 vs baseline	1.9 [0.5-7.6]		1.9 [0.5-7.6]	
**Femoral trochanter**				
Year 1 vs baseline	0.6 [0.2-1.9]	.25	0.6 [0.2-1.9]	.24
Year 3 vs baseline	1.7 [0.5-5.7]		1.7 [0.5-5.7]	
**Women[d]**				
**Variable**	**Basic model**		**Multivariable-adjusted model**	
**Bone variable**	**Intervention Group**	**Overall P-value[e]**	**Intervention Group**	**Overall P-value[e]**
**Total femur**				
Year 1 vs baseline	1.4 [0.3-5.7]	.84	1.4 [0.3-5.7]	.88
Year 3 vs baseline	1.5 [0.4-6.0]		1.4 [0.3-5.6]	
**Lumbar spines L1–L4**				
Year 1 vs baseline	0.9 [0.2-5.0]	.80	0.9 [0.2-4.8]	.80
Year 3 vs baseline	0.6 [0.1-2.8]		0.6 [0.1-2.8]	
**Femoral trochanter**				
Year 1 vs baseline	2.1 [0.5-8.0]	.54	2.1 [0.5-8.1]	.53
Year 3 vs baseline	1.1 [0.3-4.3]		1.1 [0.3-4.1]	

© 2025 Vázquez-Lorente H et al. *JAMA Network Open*.

Abbreviations: BMD, bone mineral density. CI, confidence interval. OR, odds ratio.

[a]Two-level logistic mixed models were fitted with random intercepts at cluster family (as couples from the same household were randomized together) and individual participants to assess the intervention group effect on low BMD prevalence. BMD was considered low for all participants with a T-score less than − 1 (osteopenia/osteoporosis) in every BMD measure. An interaction term between the intervention group and time, age (years), sex (men/women), and recruiting center (Navarra, Mallorca, Reus, and León) were included as fixed effects in the basic model. Baseline education level (primary or less, secondary, or college), civil status (single, divorced or separated, married, widower), smoking status (current, former, or never), diabetes prevalence (yes/no), hypertension prevalence (yes/no), hypercholesterolemia prevalence (yes/no), body mass index (kg/m^2) physical activity (METs/min/day), sedentary time (h/day), use of osteoporotic drugs (yes/no), adherence to the energy-restricted Mediterranean diet (0-17 points), and daily energy intake (kcal/day) were additionally included in the multivariable-adjusted model. Those participants taking calcium and/or vitamin D medication/supplementation at all time points were excluded. Sex (men/women) was excluded as a covariate in the analyses stratified for sex. The control group was considered as 1 (reference). Data are presented as OR [95% CI]. Significance was set at P-values ≤.05.

[b]Overall, n=736; Control group, n=369; Intervention group, n=367.

[c]Men, n=431; Control group, n=217; Intervention group, n=214.

[d]Women, n=305; Control group, n=152; Intervention group, n=153.

[e]P-value for differences intergroups.

[f]P-value represents the intervention group effects assessed for the overall follow-up period.

© 2025 Vázquez-Lorente H et al. *JAMA Network Open*.

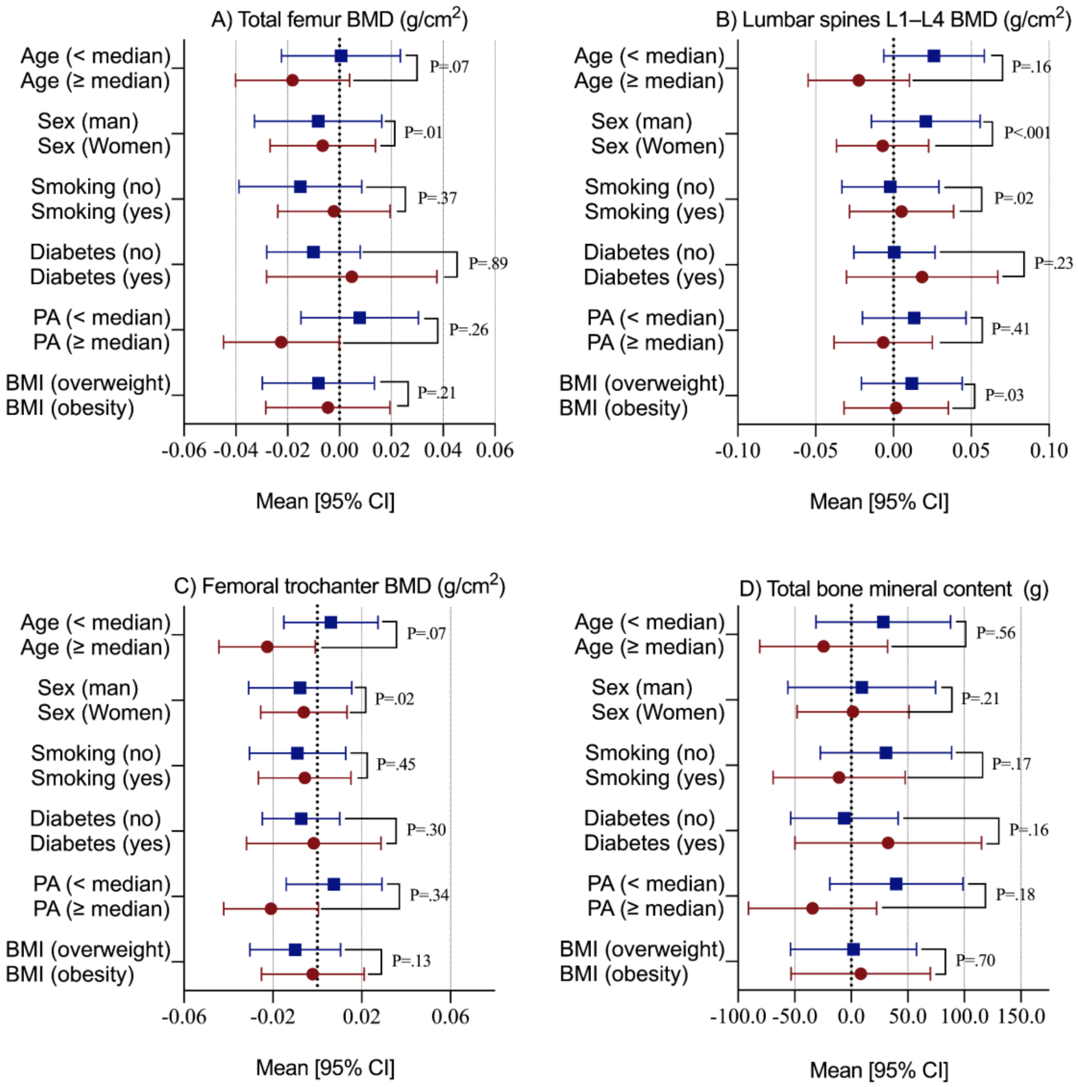

eFigure. Interaction for the BMD Variables and Total BMC With Relevant Baseline Variables of the Study

Abbreviations: BMC, bone mineral content. BMD, bone mineral density. BMI, body mass index. CI, confidence interval. PA, physical activity. Two-level linear mixed models were fitted with random intercepts at cluster family (as couples from the same household were randomized together) and individual participants to assess intervention group effects on changes in BMD variables and total BMC measured repeatedly over time (at each follow-up visit and for the overall follow-up period). An interaction term between the intervention group and time, age (years), sex (men/women), recruiting center (Navarra, Mallorca, Reus, and León), baseline education level (primary or less, secondary, or college), civil status (single, divorced or separated, married, widower), smoking status (current, former, or never), diabetes prevalence (yes/no), hypertension prevalence (yes/no), hypercholesterolemia prevalence (yes/no), body mass index (kg/m^2), physical activity (METs/min/day), sedentary time (h/day), calcium and/or vitamin D medication/supplementation (yes/no), use of osteoporotic drugs (yes/no), adherence to the energy-restricted Mediterranean diet (0-17 points), and daily energy intake (kcal/day) were included as fixed effects in the multivariable-adjusted models. An interaction term between time, intervention group, and each potential effect modifier was included within multivariable-adjusted models. Data are presented as mean [95% CI]. Significance was set at P-values ≤.05.

© 2025 Vázquez-Lorente H et al. *JAMA Network Open*.
