# Supplementary material for: Mediterranean Diet, Physical Activity, and Bone Health in Older Adults: A Secondary Analysis of a Randomized Clinical Trial
Source: JAMA Netw Open. 2025 Apr 8;8(4):e253710. doi: 10.1001/jamanetworkopen.2025.3710 (PMC11979728; doi:10.1001/jamanetworkopen.2025.3710)
Supplement: Supplement 3. — Data Sharing Statement [file jamanetwopen-e253710-s003.pdf]

# Data Sharing Statement

Vázquez-Lorente. Mediterranean Diet, Physical Activity, and Bone Health in Older Adults. *JAMA Netw Open*. Published April 08, 2025. doi:10.1001/jamanetworkopen.2025.3710

**Data**
**Additional Information:** The trial was registered in 2014 on the International Standard Randomized Controlled Trial registry [ISRCT; www.isrctn.com/ISRCTN89898870]. Trial registration number: ISRCTN89898870
**Data available:** Yes
**Data types:** Deidentified participant data
**How to access data:** There are restrictions on the availability of data for the PREDIMED-Plus study, due to the signed consent agreements around data sharing, which only allow access to external researchers for research following the project purposes. Requestors wishing to access the PREDIMED-Plus trial data used in this study can request it to the PREDIMED-Plus trial Steering Committee: predimed_plus_scommittee@googlegroups.com
**When available:** With publication

**Supporting Documents**
**Document types:** None

**Additional Information**
**Who can access the data:** To external researchers for research following the project purposes. Requestors wishing to access the PREDIMED-Plus trial data used in this study can request it to the PREDIMED-Plus trial Steering Committee: predimed_plus_scommittee@googlegroups.com.
**Types of analyses:** Specified purpose
**Mechanisms of data availability:** After approval of a proposal
